# TGFβ Inhibition during Radiotherapy Enhances Immune Cell Infiltration and Decreases Metastases in Ewing Sarcoma

**DOI:** 10.1158/2767-9764.CRC-24-0346

**Published:** 2025-08-27

**Authors:** Jessica D. Daley, Elina Mukherjee, David Ferraro, A. Carolina Tufino, Nathanael Bailey, Shanthi Bhaskar, Nivitha Periyapatna, Ian MacFawn, Sean Hartwick, Sheryl Kunning, Cynthia Hinck, Tullia C. Bruno, Adam C. Olson, Linda M. McAllister-Lucas, Andrew P. Hinck, Kristine Cooper, Riyue Bao, Anthony R. Cillo, Kelly M. Bailey

**Affiliations:** 1 Department of Pediatrics, University of Pittsburgh School of Medicine, Pittsburgh, Pennsylvania.; 2 UPMC Hillman Cancer Center, Pittsburgh, Pennsylvania.; 3 Department of Pathology, University of Pittsburgh School of Medicine, Pittsburgh, Pennsylvania.; 4 Rangos Research Center Animal Imaging Core, UPMC Children’s Hospital of Pittsburgh, Pittsburgh, Pennsylvania.; 5 Department of Immunology, University of Pittsburgh School of Medicine, Pittsburgh, Pennsylvania.; 6 Department of Structural Biology, University of Pittsburgh School of Medicine, Pittsburgh, Pennsylvania.; 7 Department of Radiation Oncology, University of Pittsburgh School of Medicine, Pittsburgh, Pennsylvania.; 8 UPMC Hillman Cancer Center Biostatistics Facility, Pittsburgh, Pennsylvania.; 9 Department of Medicine, University of Pittsburgh, School of Medicine, Pittsburgh, Pennsylvania.; 10 Center for Systems Immunology, University of Pittsburgh, Pittsburgh, Pennsylvania.

## Abstract

Ewing sarcoma is an aggressive cancer diagnosed in adolescents and young adults. Inhibition of TGFβ is being tested in limited clinical trials for relapsed Ewing sarcoma. TGFβ is an immunosuppressive cytokine that exists in latent and active states. The functional impact of TGFβ inhibition on the Ewing tumor microenvironment (TME) and on Ewing tumor behavior remains largely unknown. In this study, we use single-cell RNA sequencing analysis of human Ewing tumors to demonstrate that immune cells are the largest contributors of *TGFB1* expression in the human Ewing TME. We utilize a humanized mouse model of Ewing sarcoma to demonstrate that TME signatures in these models differ significantly from Ewing sarcoma tumors developed in immunodeficient mice. Using this humanized model, we investigate the effect of TGFβ inhibition on the Ewing sarcoma TME during radiotherapy, a treatment that is commonly used to treat unresectable, metastatic, and relapsed/refractory Ewing sarcoma that is known to enhance TGFβ activation in multiple cancers. Utilizing a trivalent ligand TGFβ trap to inhibit TGFβ, we demonstrate that in combination with radiotherapy, TGFβ inhibition both increases Ewing sarcoma immune cell infiltration and decreases lung metastatic burden *in vivo*. These data demonstrate the value of immunocompetent models to address immune-biological preclinical questions in Ewing sarcoma and demonstrate that TGFβ inhibition during radiotherapy is a promising strategy to enhance antitumor immune response and improve treatment efficacy for metastatic Ewing sarcoma.

**Significance:**

This work demonstrates the importance of disrupting immunosuppression during radiotherapy to reduce lung metastatic potential in Ewing sarcoma. Humanized mouse models of Ewing sarcoma are also established as an immunocompetent preclinical tool to ask therapeutic questions about the Ewing TME.

## Introduction

The tumor microenvironment (TME) is a dynamic, interconnected network of tumor cells and other non-malignant supporting elements such as stromal cells, immune cells, and cytokines (1). Tumor treatment, such as chemotherapy and radiation, remodels the TME by promoting tumor cell apoptosis, altering the cytokine profile, shifting the composition of tumor immune infiltrates, and driving functional dysregulation of immune cells (2–4). The TME can support and promote tumor cell growth, metastases, and survival and paradoxically, in some cases, tumor treatment enhances the tumor-promoting effects of the TME (5–8). Emerging cancer therapeutics are designed to remodel the TME to reduce immunosuppression and promote tumor control (9, 10).

Recent studies of the TME of Ewing sarcoma, an aggressive fusion oncoprotein (EWSR1::FLI1)–driven primary bone tumor of adolescents and young adults (11–13), revealed the importance of extracellular matrix (ECM) remodeling (14) and hypoxia (15, 16) in promoting Ewing sarcoma tumor cell survival and metastatic potential. An aspect of the Ewing sarcoma TME that continues to be poorly understood (and not well represented in *in vivo* studies of Ewing sarcoma) is immunobiology. This, in part, is due to the historical lack of syngeneic or transgenic (immunocompetent) mouse models of this cancer. Despite international efforts over decades to develop a transgenic mouse model of Ewing sarcoma (17, 18), attempts to induce the expression of EWSR1::FLI1 at the right level, in the right cell, and in the correct developmental state have proved very challenging and ultimately unsuccessful. Tanaka and colleagues (19) generated a mouse Ewing sarcoma tumor model by expressing EWSR1::FLI1; however, scalability of this approach is technically challenging. In 2022, Vasileva and colleagues (20) developed an immunocompetent zebrafish model of Ewing sarcoma; characterization of the tumor immune microenvironment of this model has not yet been explored.

Our current understanding of the Ewing sarcoma tumor immune microenvironment is largely derived from the analysis of limited human patient samples. Primary, pretreatment Ewing sarcoma tumor formalin-fixed, paraffin-embedded (FFPE) specimens from the bone demonstrate an overall low immune infiltrate (21). Recent work from our laboratory utilized fresh tumors and paired peripheral blood specimens from patients with Ewing sarcoma to conduct a focused single-cell RNA sequencing (scRNA-seq) analysis of CD45^+^ cells (i.e., all cells of hematopoietic origin) infiltrating tumors. We demonstrated increased immune cell infiltration at the time of relapse in comparison with the time of original diagnosis in Ewing sarcoma. Furthermore, intracellular communication analyses identified CD14^+^CD16^+^ macrophages as drivers of immune infiltration. We found that, in contrast to our parallel analyses of osteosarcomas, the mechanism of macrophage-driven immune infiltration in Ewing sarcoma was less dependent on CXCL10/CXCL12 than in osteosarcoma (22). Recent additional studies utilizing scRNA-seq of human Ewing sarcomas identify immunosuppressive myeloid cell populations as a predominant feature (23, 24). The specific factors in the TME that modulate Ewing sarcoma tumor immunobiology, including cytokine expression, remain largely unknown.

In the current study, we demonstrate that immune cells are the largest contributors of *TGFB1* in the human Ewing sarcoma TME. TGFβ is an immunosuppressive cytokine that promotes regulatory T cells and pro-tumorigenic macrophages (25, 26). We developed and utilized a CD34^+^ humanized mouse model of Ewing sarcoma (human immune cells plus human tumor cells) to study the impact of TGFβ inhibition, utilizing a TGFβ trap, on Ewing sarcoma biology. As radiotherapy is a treatment modality commonly used for the treatment of metastatic and relapsed Ewing sarcoma (27–29) and is known to promote latent TGFβ activation, we also investigate the impact of TGFβ inhibition specifically during radiotherapy. To understand immune contexture–dependent contributions, we conducted parallel analyses of identical Ewing tumors in immunodeficient NOD/SCID gamma (NSG) mice and found that Ewing sarcoma tumors developed in an immunocompetent, humanized murine model have increased expression of important modulators of the TME, including *TGFB1*, and increased metastatic potential, as compared with tumors developed in NSG, immunodeficient models. Inhibition of TGFβ during radiotherapy in our humanized mouse model of Ewing sarcoma both increases immune cell infiltration and decreases lung metastases. These data demonstrate that TGFβ signaling modulates both immune infiltration and metastasis in Ewing sarcoma and provide early preclinical rationale for combining TGFβ inhibition with radiotherapy for the treatment of aggressive Ewing sarcoma. More broadly, we demonstrate the utility of immunocompetent Ewing sarcoma models as a tool to enhance the preclinical testing of TME-modulating agents in Ewing sarcoma.

## Materials and Methods

### Cell lines

TC32 (Childhood Cancer Repository, cccells.org) and A673 (ATCC) Ewing sarcoma cell lines were cultured in glutamine containing phenol-free RPMI supplemented with 10% FBS. Cell line identification was authenticated by short tandem repeat profiling at the University of Arizona Genetics Core. *Mycoplasma* contamination was monitored using the MycoAlert PLUS Mycoplasma Detection Kit (Lonza, cat#. LT07-701). HLA-A2 status of Ewing sarcoma tumor cell lines was determined.

### Mouse models and establishment of Ewing sarcoma tumors

Protocols for *in vivo* mouse experiments were approved by the Institutional Animal Care and Use Committee at the University of Pittsburgh under the reference #22010361. Hu-CD34^+^ mice were purchased from The Jackson Laboratory [strain number 705557, The Jackson Laboratory, NSG strain (005557) and selected “with CD34^+^ engraftment” on the hu-mice order form]. Immunodeficient NSG mice were also purchased from The Jackson Laboratory (strain number 005557). For establishment of tumors in both the Hu-CD34^+^ mice and the NSG mice, 5 × 10^5^ TC32 or A673 Ewing sarcoma cells [mixed in a 1:1 solution of cold growth factor–reduced Geltrex (Life Technologies, cat. #A1413202) with PBS] were injected into the mouse in a para-tibial location, an injection method previously described for the study of bone sarcomas in murine models (30).

### Tumor treatment with radiotherapy

Lab members completed radiation safety training at The University of Pittsburgh. Mice bearing Ewing sarcomas received a single dose (fraction) of 4 or 5 Gy (dose determined by prior *in vitro* testing) using an X-RAD 320 (Precision X-Ray) using Filter 2 (1.5 mm Al + 0.25 mm Cu +0.75 mm Sn). A full-body lead shield with window to expose the lower extremity (Precision X-Ray, part number XD1907-2021) was used to isolate the para-tibial tumors for focal radiotherapy and minimize off-tumor effects. Treatment was delivered between 20 and 22 days following tumor injection, when the average tumor width was 5 mm.

### Live-cell monitoring and apoptosis assays

Cells were seeded into 96-well plates (Corning 3610 or 3596) in 100 mL of Fluorobrite media (Gibco, #A18967-01) containing 5% FBS minimally. The cells were seeded at a starting cell count of 5,000 to 10,000 cells per well. Cells (A673 or TC32) were treated with either 5, 2, or 0 Gy. IncuCyte Caspase 3/7 green reagent (Essen BioScience, cat. #4440) was added to a final dilution of 1:1,000. Phase-contrast images of the cells in standard culture conditions were obtained at 3- to 6-hour intervals using an IncuCyte S3 or IncuCyte Zoom (Essen BioScience). Green fluorescence images were additionally captured for apoptosis assays. Experiments were repeated minimally in technical and biologic triplicates. Relative apoptosis was determined by normalizing the green count to the cell confluence.

### Mouse treatment with RII-BG_E_-RII and plasma testing of TGFβ inhibition

Endotoxin-free RII-BG_E_-RII (RER; a trivalent ligand trap for TGFβ) was prepared (31) and delivered at a dosage of 50 μg per day via intraperitoneal injection, as previously described (32). Control mice received intraperitoneal injection of PBS (50 mL daily). Treatment was started 20 to 22 days after tumor cell injection when the average tumor width was 5 mm. Treatment with either RER or PBS (control) started on day 1 of radiotherapy. For immunobiology studies, a total of six RER/PBS doses were delivered (mice euthanized on day 7). For tumor growth and survival studies, daily RER/PBS doses continued until experimental end points were reached. See individual figures for additional details. Plasma isolated from mouse peripheral blood at the time of euthanasia was analyzed using ELISA (Abcam, cat. #ab1006447) for quantification of TGFβ1.

### FFPE preparation and IHC

At the time of euthanasia (see individual figures for timing details), tumors and lungs were collected. For tissue histology, a portion of the tumor, as well as both lungs, was placed in formalin for at least 24 hours prior to paraffin embedding. The University of Pittsburgh Pitt Biospecimen Core acquired 5-micron serial sections (interval of 500 microns) of the blocks and performed hematoxylin and eosin staining. This was based on prior literature (33), and in consultation with the Pitt Biospecimen Core, the University of Pittsburgh Medical Center Department of Pathology clinical IHC protocol was used for CD99 and NKX2.2 staining (markers of Ewing sarcoma).

### Examination of lung metastases

Serial lung sections (five slides per mouse) were reviewed by an independent pathologist blinded to the treatment conditions, as previously described (33). Bilateral lungs were examined. Independent metastatic lesions on each slide were counted as a single metastatic focus.

### Tumor imaging and volume calculations

Mice underwent imaging of bilateral lower extremities weekly, starting 1 week following tumor cell injection. Animals were anesthetized with 1.5% isoflurane in oxygen, administered via a nose cone, and positioned prone on a 38-mm imaging bed. Each mouse was gently secured at the torso and legs with low radiodensity micropore tape to minimize movement during scanning. Respiration was monitored continuously throughout the procedure. Scans were acquired using a Siemens Inveon Multi-Modality microPET/SPECT/CT system (Siemens Medical Solutions) at 80 kV and 500 μA, with an exposure of 1,400 ms. A transaxial field of view of 40.78 mm and an axial field of view of 75.74 mm with 2 × 2 binning offered a resolution of 45.52 μm. An aluminum filter was used to minimize beam hardening. A total of 220 projections were collected over a 180 degrees rotation, with a rotation step size of 0.818 degrees and a settle time of 0 ms per projection, resulting in a scan time of approximately 13 minutes per animal. Image reconstruction was performed using a Feldkamp algorithm, with no downsampling, application of a Shepp–Logan filter, and slight noise reduction. Beam hardening correction was applied using the “mouse” preset available in the Inveon reconstruction software. The reconstructed datasets were used to visualize and assess tumor volume, shape, and spatial relationship with surrounding bone. Image processing and segmentation were conducted using Inveon Research Workplace, enabling high-resolution, non-invasive evaluation of tumor progression *in vivo*.

### Analysis of mouse survival

Mouse survival studies following treatment with ± radiotherapy and ± RER were conducted. End points were defined by meeting one of the following parameters: loss of >20% body weight, primary tumor size greater than 15 mm in size in any diameter, natural death, or reaching a time point of 8 weeks following tumor cell injection (experiment termination).

### Blood and tumor processing and flow cytometry analysis

Blood was collected from mice at the time of euthanization. Whole blood was spun at 400 × G for 10 minutes with brakes off. Plasma was collected and immediately stored at −80°C for future analysis. Red cell lysis was performed on the remaining cell pellet using BD Pharmlyse buffer (BD Biosciences, cat. #555899) per the manufacturer’s instructions. The remaining cell pellet was washed and resuspended in PBS.

Mouse tumors were manually dissected into 1-mm^2^ pieces with a scalpel and incubated for 15 minutes at 37°C in 5% CO_2_ in 5 mL of serum-free, phenol-free RPMI and 50 mg/mL Liberase DL (MilliporeSigma, cat. #5466202001). Single-cell suspensions were then passed over a 70-μm filter to remove debris. Both peripheral blood mononuclear cells and tumor cell suspensions were stained with conjugated antibodies for 20 minutes in the dark. The following antibodies were used for surface staining: human CD163 BV650 1:100 dilution (BD Biosciences, 563888), human CD25 BV711 1:200 dilution (BioLegend, 302636), human CD20 BV750 1:200 dilution (BD Biosciences, 747062), human CD8 BUV496 1:200 dilution (BD Biosciences, 612942), human CD45 BUV395 1:300 dilution (BD Biosciences, 563792), human CD14 BUV737 1:300 dilution (BD Biosciences, 612763), human CD4 BUV563 1:400 dilution (BD Biosciences, 741353), human CD103 BUV615 1:400 dilution (BD Biosciences, 751285), human CD3 Spark Blue 550 1:100 dilution (BioLegend, 344852), human CD11b PerCP/Cyanine5.5 1:100 dilution (BioLegend, 301328), CD56 human CD19 APC/Fire 810 1:100 dilution (BioLegend, 302272), human HLA-DR Alexa Fluor 488 1:300 dilution (BioLegend, 307620), human CD16 PE/Dazzle 594 1:400 dilution (BioLegend, 302054), human CD33 PE 1:800 dilution (BioLegend, 366608), human Ki67 BV421 1:200 dilution (BioLegend, 350506), and human FoxP3 EF450 1:100 dilution (Invitrogen, 48477642). Cells were stained with the viability dye Zombie NIR Fixable Viability Kit (BioLegend, cat. #423105). Stained cells were fixed using eBioscience Fixation/Permeabilization Concentration (Invitrogen, cat. #00-5123-43). Stained cells were analyzed using a Cytek Aurora spectral flow cytometer and data were analyzed using the FlowJo software (v10.9.0). Additional analysis was performed utilizing Cytobank Software (beckman.com).

### Tumor RNA extraction and bulk RNA sequencing

A portion of tumors isolated from mice at sacrifice was flash frozen using liquid nitrogen. RNA was isolated from the tumors using the Qiagen QIAshredder (Qiagen, cat. #79656) and Qiagen RNeasy Plus Micro Isolation Kit. (Qiagen, cat. #74034). RNA concentration was measured using NanoDrop (Thermo Fisher Scientific). mRNA library preparation and sequencing were performed at the Health Sciences Sequencing Core at Children’s Hospital of Pittsburgh with a 2 × 50 paired end read length and 40 million reads per sample.

### RT-PCR

Utilizing the RNA isolated for bulk RNA sequencing (RNA-seq) above, cDNA synthesis was performed on 1 μg of RNA with the High-Capacity cDNA Reverse Transcription Kit (Thermo Fisher Scientific, cat. #4374966) and an Applied Biosystems Veriti 96-well Thermocycler. qRT-PCR analysis was performed using Taqman probes (Life Technologies; *EWSR1::FLI1* Hs03024497_ft and *RPLP0* Hs00420895_gH) and Taqman Universal PCR Master Mix (Life Technologies, cat. #4304437) and the StepOnePlus Real-Time PCR system (Life Technologies).

### RNA-seq library generation

RNA was assessed for quality using an Agilent TapeStation 4150/Fragment Analyzer 5300 and RNA concentration was quantified on a Qubit FLEX fluorometer. Libraries were generated with the Illumina Stranded mRNA Library Prep kit (Illumina, 20040534) according to the manufacturer’s instructions. Briefly, 1,000 ng of input RNA was used for each sample. Following adapter ligation, 10 cycles of indexing PCR were completed, using IDT for Illumina RNA UD Indexes (Illumina, 20040553-6). Library quantification and assessment were done using a Qubit FLEX fluorometer and an Agilent TapeStation 4150/Fragment Analyzer 5300. Libraries were normalized and pooled to 2 nmol/L by calculating the concentration based off the fragment size (base pairs) and the concentration (ng/μL) of the libraries.

### Library sequencing

Sequencing was performed on an Illumina NextSeq 2000, using a P3 100 flow cell. The pooled library was loaded at a concentration of 750 pmol/L and sequencing was carried out with read lengths of 2 × 58 bp, with a target of 40 million reads per sample. Sequencing data were demultiplexed by the on-board Illumina DRAGEN FASTQ Generation software (v3.10.12).

### Bulk RNA-seq gene expression quantification

After quality control, the paired-end reads were pseudoaligned to the human reference transcriptome (GRCh38) with Gencode annotation (v28) and summarized at transcript level using kallisto (v0.48.0). Transcript abundance was summarized into gene level using tximport (v1.22.0) and was trimmed mean of M-values normalized and log_2_-transformed. Genes with low expression (counts per million of mapped reads < 3) were removed prior to normalization and statistical comparison. Gene lists of comparison groups are included in Supplementary Table S1.

### Human Ewing sarcoma tumor processing for scRNA-seq

Viably preserved Ewing tumor biopsy specimens were rapidly thawed, processed into single-cell suspensions, and immediately loaded into the 10X Genomics Controller as per the manufacturer’s protocol (as previously described; ref. 22) to analyze gene expression signatures of individual cells within the TME (Institutional Review Board approved STUDY19030108, all participants provided consent on the basis of Helsinki principles). 10x Genomics library preparation was conducted at The University of Pittsburgh Single Cell Core.

### Processing of scRNA-seq data

Following sequencing, raw sequencing files were demultiplex into FASTQ files using bcl2fastq (v.2.20.0) and were aligned to the reference genome GRCh38 using CellRanger (v6.0.1). Following alignment, feature/barcode matrices were read into Seurat (v4.3.0.1) in R (4.2.0). Previously described data from patients with Ewing sarcoma (22) were also read into Seurat and a unified analysis object was created. Cells with fewer than 200 genes per cell or with >10% of reads aligning to mitochondrial genes were excluded as part of a quality control workflow.

### Dimensionality reduction and clustering

Dimensionality reduction was performed in Seurat as previously described (22) with the addition of an integration workflow. Briefly, the top 2,000 highly variable genes were identified in each sample and SCTransform was used to normalize expression values in each sample individually. Next, anchors were identified, and data integration was performed to reduce technical variation between samples. Following integration, principal component analysis was performed using the 2,000 integrated features. Consecutive principal components were heuristically selected based on the variance explained per principal component, and the selected principal components were used for the generation of Uniform Manifold Approximation and Projections (UMAP) and as input for Leiden-based clustering.

### Identification of cell types

Cell types were identified as previously described (22). Briefly, cell types were identified based on the expression of canonical lineage markers including for leukocytes (lymphocytes including T-cell subsets, B cells, and NK cells; myeloid cells including monocytes, macrophages, and dendritic cell) tumor populations, osteoclasts, and fibroblasts using markers *PTPRC*, *CD3D*, *CD4*, *CD8A*, *FOXP3*, *CD14*, *FCGR3A*, *MS4SA1*, *CD1C*, *IL3RA*, *MMP9*, and *COL1A1*. Lymphocyte subsets were further refined by performing dimensionality reduction and clustering on only lymphocyte subsets.

### Differential gene expression detection and pathway enrichment analysis

Differentially expressed genes (DEG) between groups of interest were identified using the Linear Models for Microarray and RNA-Seq Data (limma) voom algorithm with precision weights (v3.50.3) and filtered by FDR-adjusted *P* < 0.05 and fold change ≥1.5 or ≤ −1.5. Pathways enriched in significant DEGs were identified using Enrichr with BioPlanet2019 (accessed August 2023).

### Causal network analysis

Upstream transcriptional regulators and the change of direction (activation or inhibition) were predicted from target molecules (encoded by significant DEGs) using Ingenuity Pathway Analysis (IPA; Qiagen) with the curated Ingenuity Knowledge Base (accessed August 2023) and filtered at overlap *P* < 0.05 (measuring the enrichment of target molecules in the dataset) and *z*-score ≥1.95 (measuring the predicted activation level of the pathways).

### Digital immune cell population deconvolution

scRNA-seq count matrix and cell type annotations previously published by our group (21) were used to construct gene signature matrix using cibersortx with 1,000 permutations and default parameters. The signature was subsequently used for estimating the fraction of immune cell populations in bulk RNA-seq data.

### Statistical analysis of RNA-seq data

A hypergeometric test was used for pathway enrichment detection. A nonparametric Wilcoxon test was used to compare cell population fractions between groups. Benjamini–Hochberg–adjusted FDR was used for adjustment of multiple comparisons. All tests are two-sided unless otherwise noted. FDR was controlled at 0.05 level.

### Statistical analysis

To determine differences in total human CD45^+^ cell counts, a linear model was fit with a fixed effect for treatment group and a random effect for biological replicate. Pairwise differences compared with control only were evaluated using degrees of freedom based on the Satterthwaite method. To determine difference in serum levels of TGFβ1, a paired *t* test was performed utilizing Graphpad Prism v.10. To determine the difference in lung metastatic burden, the correlation of counts across slides was adjusted by calculating the average number of lung metastases across all five slides for each mouse. Lung samples collected at a fixed time point were analyzed separately from samples collected at variable times of sacrifice. Lung samples collected at various times (when experimental end points of tumor growth and survival were met) were analyzed stratified by time before or after median time to collection. A Poisson model was fit to the data to model the average count of lung metastases across treatment groups. Tumor growth curves were estimated by a linear mixed model with a fixed effect for treatment and random effect for mouse. Measurements were log-transformed prior to model fit to achieve linearity, and model estimates were converted back to the original scale to be displayed with the observed data. Animals with missing tumor volume measurements after the start of treatment were excluded from modeling. All animals were included in the survival models and other end points. Statistical analysis was performed using RStudio (v 2023.09.1).

### Data availability

The raw and processed RNA-seq data have been deposited in the Gene Expression Omnibus database under GSE261693 and GSE198896. Additional data will be provided upon request.

### Software for schematics

Schematics were created using biorender.com.

## Results

### *TGFB1* expression in human Ewing sarcoma occurs predominantly in the immune cell compartment

TGFβ is an immunosuppressive cytokine that prevents effective antitumor immune responses and promotes remodeling of the ECM toward a pro-tumor phenotype (13, 14). Increased TGFβ pathway signaling has been noted in Ewing sarcoma tumors that demonstrate a pro-metastatic, aggressive phenotype (34). Recent work also demonstrates that TGFβ is elevated in the serum of patients with Ewing sarcoma as compared with healthy controls (35). Strategies to investigate the immunosuppressive effect of TGFβ specifically on NK-cell therapy are currently being tested clinically in patients with relapsed Ewing sarcoma (NCT05634369; ref. 36).

**Figure 1:**
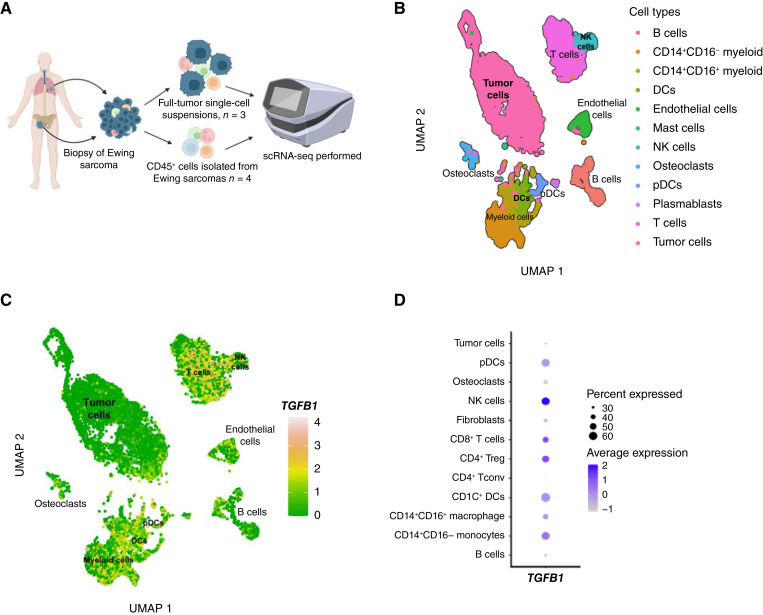
*TGFB1* is predominantly expressed by immune cell populations in Ewing sarcoma. **A,** Experimental overview. **B,** Uniform Manifold Approximation and Projection embedding of 17,610 cells from Ewing sarcoma tumors from patients. **C,** Expression of *TGFB1* across immune, non-immune, and tumor populations from the seven patients shown in **B**. Log_2_ expression levels of *TGFB1* are visualized across all cells. Inferred cell types from all cells and patients are shown. *TGFB1* is expressed across numerous lymphocyte (CD4^+^ regulatory T cells, CD8^+^ T cell, and NK cells) and myeloid cell populations (monocytes, macrophages, and dendritic cells) in the Ewing sarcoma TME but is generally not expressed by non-immune lineages. **D,** Dot plot showing the mean *TGFB1* expression levels (colors of the dot) and the frequency of cells that express *TGFB1* (size of the dot) across all cells from the TME. DC, dendritic cells; pDC, plasmacytoid dendritic cells; Treg, regulatory T cells; UMAP, Uniform Manifold Approximation and Projection. [**A,** Created in BioRender. Daley, J. (2025) https://BioRender.com/27tjakt.]

The contribution of individual cell types in the Ewing sarcoma TME to TGFβ expression is poorly understood. Thus, we sought to analyze *TGFB1* expression in human Ewing sarcomas on a single-cell level. We conducted scRNA-seq on fresh (22) and viably preserved human Ewing tumors acquired through an Institutional Review Board–approved protocol (Fig. 1A). Given the overall low percentage of immune cells infiltrating human Ewing sarcomas (37), some samples were specifically enriched for CD45^+^ cells only to ensure representation of broad immune cell types (previously published by our group; ref. 22). We also analyzed an additional three Ewing sarcomas by scRNA-seq and included all cell types (tumor cells, immune cells, and stromal cells; see Supplementary Table S2). Resulting cell clusters were identified based on transcriptomic profiles (Fig. 1B). The number of cells in each cluster is included in Supplementary Table S3. We then queried the expression of *TGFB1* and found that *TGFB1* is most highly expressed by immune cell populations in human Ewing sarcomas, with low expression in tumor and stromal cell populations (Fig. 1C). To account for the differences in samples in which CD45^+^ cells were specifically isolated versus samples in which all cell types were analyzed, we also generated Uniform Manifold Approximation and Projections demonstrating *TGFB1* expression in these groups separately (Supplementary Fig. S1). *TGFB1* expression is highest in NK cells, CD4^+^ T regulatory cells, CD8^+^ T cells, and CD14^+^CD16− monocytes isolated from human Ewing tumors (Fig. 1D). We also determined the expression of other TGFβ isoforms, *TGFB2* and *TGFB3*, in the immune compartment of Ewing sarcomas and found that *TGFB1* is the predominant isoform expressed (Supplementary Fig. S2). These findings demonstrate that *TGFB1* is expressed almost exclusively in the immune cell compartment of Ewing sarcoma and highlight that rigorous studies of TGFβ in the Ewing sarcoma TME require an immunocompetent model.

### Ewing sarcomas developed in an immunocompetent, humanized hu-CD34^+^ model are biologically and transcriptionally distinct from Ewing sarcoma developed in an immunodeficient mouse model

As a next step toward elucidating the functional role of TGFβ in the Ewing sarcoma TME, we developed and validated an immunocompetent mouse model of Ewing sarcoma utilizing hu-CD34^+^ humanized mice. The hu-CD34^+^ murine model is derived from an immunocompromised NSG mouse (lacking mature T cells, B cells, and functional NK cells) that undergoes transplantation utilizing human CD34^+^ cord blood–derived stem cells after total body irradiation conditioning (38, 39) resulting in reconstitution with CD45^+^ human immune cell lineages including B cells, T cells, and myeloid cells (Supplementary Fig S3). Unlike humanized models utilizing peripheral blood mononuclear cells for immune cell reconstitution, the CD34^+^ model is much less prone to the development of GVHD, and no evidence of GVHD was noted throughout the duration of our tumor experiments.

**Figure 2:**
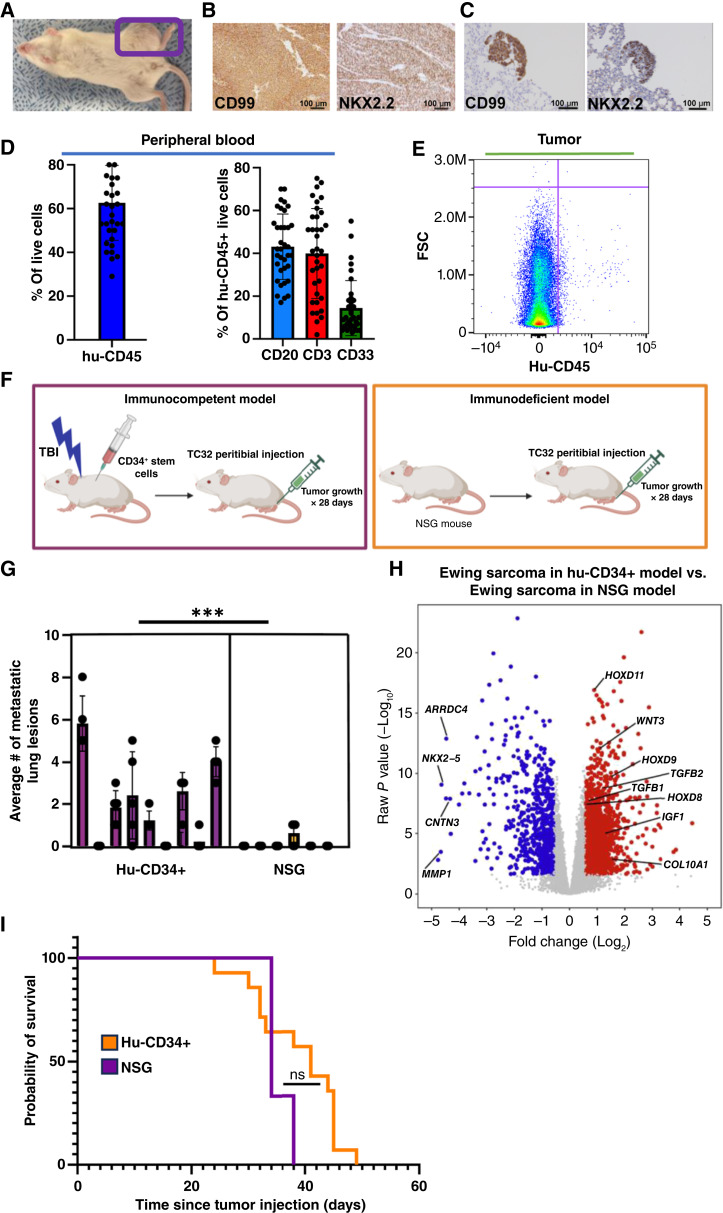
Ewing sarcomas developed in the hu-CD34^+^ model are biologically and transcriptionally distinct from tumors developed in an immunodeficient model. **A,** Orthotopic Ewing sarcomas are successfully established in a humanized (hu-CD34^+^) mouse. **B,** Representative CD99 and NKX2.2 IHC staining of Ewing sarcomas developed in a hu-CD34^+^ model, shown with a magnification of 200×. **C,** Representative CD99 and NKX2.2 IHC staining of lung metastases that developed in the hu-CD34^+^ Ewing sarcoma model, shown with a magnification of 200×. **D,** Immunophenotyping performed on peripheral blood mononuclear cells obtained from mice bearing TC32 Ewing sarcoma tumors at euthanasia following tumor growth demonstrates persistence of engrafted human immune cells. HuCD45 represents % of all peripheral lymphocytes that are huCD45 positive. The remaining cells that are hu-CD45 negative represent (defective) peripheral blood mononuclear cells of mouse origin. The percent of hu-CD45 cells that express CD20, CD3, and CD33 (representing B cells, T cells, and myeloid cells, respectively) is shown. *n* = 36 mice. Error bars represent SD. **E,** Representative flow cytometry plot demonstrates low (1.24% of all cells in the tumor) baseline huCD45^+^ infiltration in Ewing sarcomas developed in hu-CD34^+^ mice. **F,** Schematic depicting hu-CD34^+^ vs. NSG mouse models used in these experiments. **G,** Bilateral lungs were harvested from hu-CD34^+^ or NSG mice harboring TC32 Ewing sarcoma tumors, and the presence of metastatic foci per section was determined by an independent pathologist. Each bar represents average number of metastatic lesions per slide in individual mouse. ***, *P* < 0.001. Error bars represent SD. A *t* test was performed. *n* = 9 humanized mice and *n* = 6 NSG mice. **H,** Volcano plot of DEGs in TC32 tumors established in hu-CD34^+^ mice vs. NSG mice. **I,** Tumors were established (TC32 cell line) in hu-CD34^+^ or NSG mice. Time from tumor injection to survival was determined using pre-specified end points for survival (see “Materials and Methods”). Kaplan–Meier estimate was used for analysis. *N* = 7 and *N* = 9 for Hu-CD34^+^ and NSG, respectively. ns = *P* value ≥ 0.05. [**F,** Created in BioRender. Bailey, K. (2025) https://BioRender.com/4jtlo4v.]

Ewing sarcoma tumors were established in an orthotopic, para-tibial location (Fig. 2A). The resulting tumors demonstrated a soft tissue component with local bone destruction, which is a classic feature of human Ewing sarcoma (40). Bony destruction in this model is demonstrated via CT scan imaging in Supplementary Fig. S4. A para-tibial method was utilized instead of methods that directly inject into the marrow space to avoid direct seeding of tumor cells into the vascular space (41). Resulting tumors were confirmed to be Ewing sarcoma with positive IHC staining for CD99, a membrane protein expressed uniformly in Ewing tumors, and nuclear staining of NK homeobox 2 (NKX2.2), a transcription factor upregulated by EWSR1::FLI1 fusion oncoprotein (Fig. 2B). Negative controls for IHC staining were included (Supplementary Fig. S5). Of note, spontaneous pulmonary metastases develop in this model (Fig. 2C), a finding which recapitulates the situation in humans, as the lung is the most common site of metastasis in patients with Ewing sarcoma (42). Peripheral blood was also collected from mice at the time of euthanasia, and we demonstrate that the human immune cells persist in the peripheral blood of these mice throughout the duration of the experiment (Fig. 2D), including NK cell populations (Supplementary Fig. S6). The remainder of live cells in the peripheral blood that are hu-CD45 negative represent mouse CD45^+^ cells present in the NSG model (dysfunctional). Immunophenotyping was also performed on single-cell suspensions of Ewing sarcomas established in the hu-CD34^+^ model, and results demonstrated low baseline infiltration of hu-CD45^+^ cells in Ewing sarcomas (Fig. 2E), similar to the level of immune infiltration noted in patient Ewing sarcomas at baseline (Supplementary Table S4; refs. 43, 44).

Having established a hu-CD34^+^ Ewing sarcoma model, we next sought to understand the transcriptomic and biological differences noted in Ewing tumors established in hu-CD34^+^ versus NSG (immunodeficient) mice. Tumors were established in both the hu-CD34^+^ immunocompetent mouse model and an NSG immunodeficient murine model utilizing the human TC32 Ewing sarcoma cell line (Fig. 2F). Tumors were allowed to develop for approximately 4 weeks prior to euthanasia, and both tumors and lungs were collected at this time. Bilateral lung tissue was formalin-fixed and paraffin-embedded and the resulting blocks underwent serial sectioning. Serial lung sections were reviewed independently by a blinded pathologist to determine the burden of lung metastases. We found significantly more lung metastases present in the orthotopic hu-CD34^+^ mice harboring TC32 Ewing sarcoma tumors as compared with NSG mice harboring TC32 Ewing sarcoma tumors (Fig. 2G). To examine transcriptional differences between Ewing tumors developed in hu-CD34^+^ versus NSG mice, bulk RNA-seq was performed on RNA isolated from flash-frozen tumors. Unsupervised clustering of the transcriptional profiles across all samples indicated that Ewing tumors developed in the hu-CD34^+^ and immunodeficient NSG models cluster separately (Supplementary Fig. S7). A total of 2,112 DEGs (fold change >1.5, *P* value < 0.05) were identified in tumors developed in the humanized model versus NSG model (Fig. 2H). Upregulated gene transcripts in Ewing sarcoma tumors developed in the hu-CD34^+^ models compared with the NSG model include *Wnt3,* Hox gene family members, and collagen family genes, some of which have been previously reported to be pro-tumorigenic in Ewing sarcoma (14). Tumors grown in hu-CD34^+^ mice demonstrated upregulation of pathways driving collagen biosynthesis and ECM remodeling (Supplementary Fig. S8) when compared with Ewing sarcoma tumors established in immunodeficient mice. We hypothesized that TGFβ1 expression would be increased in tumors developed in an immunocompetent model because our human Ewing sarcoma data demonstrate that *TGFB1* is expressed predominantly in the immune compartment (Fig. 1). Serum levels of TGFβ1 in both hu-CD34^+^ and NSG mice were analyzed by ELISA and demonstrated significantly more TGFβ1 present in the hu-CD34^+^ mouse model as compared with the NSG model (Supplementary Fig. S9). Taken together, these data demonstrate that Ewing sarcomas developed in an immunocompetent, humanized murine model have increased expression of important modulators of the TME, including TGFβ1, and increased metastatic potential as compared with tumors developed in NSG, immunodeficient models.

We found no statistically significant difference in primary tumor growth rates (as determined by weekly CT imaging; Supplementary Fig. S10) or overall survival (Fig. 2I) between the NSG and hu-CD34^+^ models.

### Transcriptional remodeling of Ewing sarcoma after radiotherapy is dependent on immune contexture

Previous investigations of epithelial-derived cancers (non-sarcomas) have demonstrated an important role for TGFβ in modulating the net response to cancer therapies (45, 46). Specifically, TGFβ signaling is well described to be increased in the TME of these epithelial cancers following radiotherapy, and when present, TGFβ can blunt the antitumor immune response. The primary mechanism by which TGFβ signaling is increased following radiotherapy is through radiotherapy-induced activation of the latent TGFβ pro-peptide present in the TME and not necessarily through increased production of TGFβ (47–49). This increase in active TGFβ is expected to occur as early as 6 hours following radiotherapy (50). Radiotherapy is an essential local control modality for patients with unresectable, metastatic, or relapsed Ewing sarcoma. Recent clinical trials have demonstrated that radiotherapy for localized Ewing sarcoma is an equivocally effective local control method for Ewing sarcoma that cannot be surgically resected (51). Despite the fact that many patients with Ewing sarcoma are treated with radiotherapy, the effect of radiotherapy on the tumor immune microenvironment of Ewing sarcoma is not well understood.

**Figure 3:**
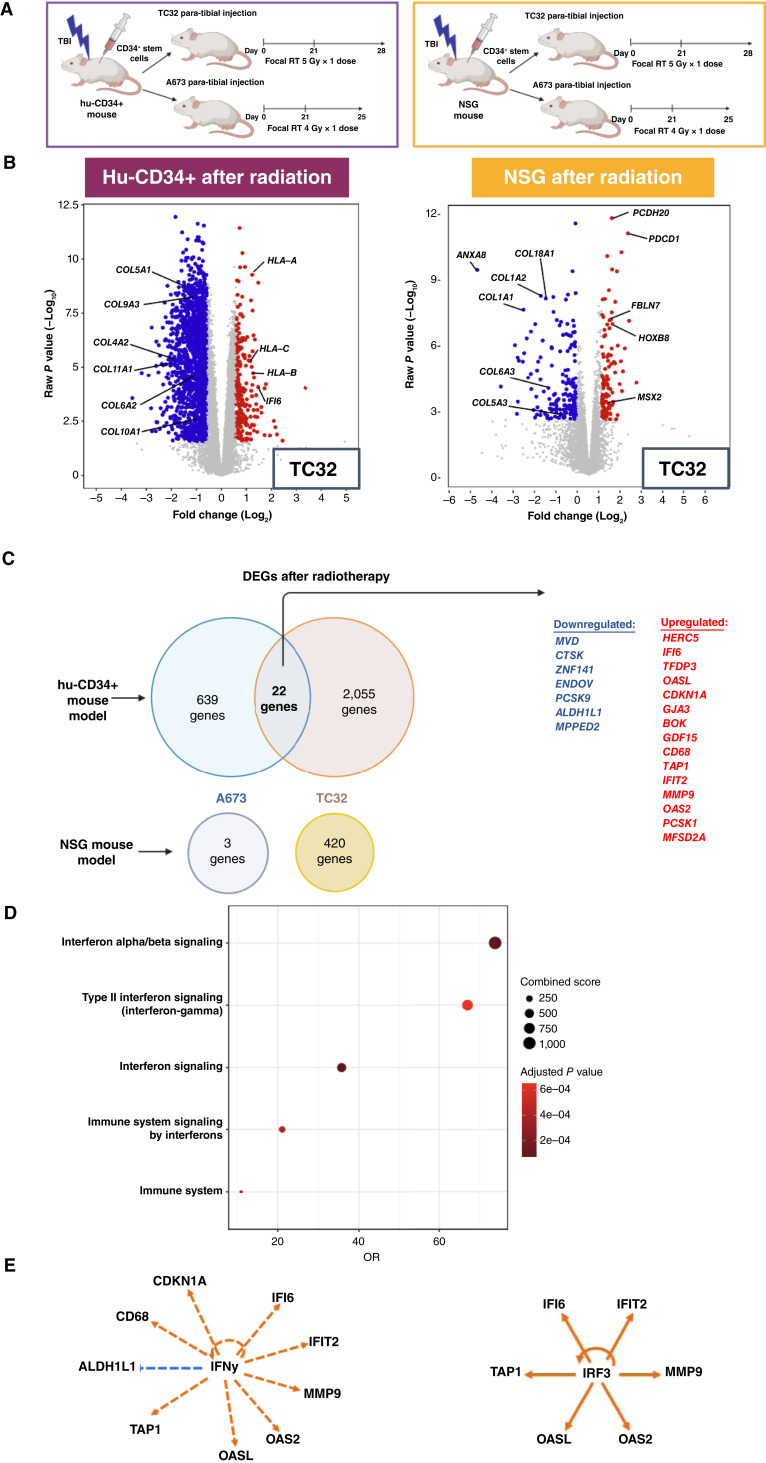
Radiotherapy modulates the Ewing sarcoma transcriptional profile in an immune contexture–dependent manner. **A,** Experimental overview of Ewing sarcomas established in hu-CD34^+^ and NSG mouse models followed by treatment with radiotherapy. **B,** RNA was extracted from tumors at the experiment termination and bulk RNA-seq was performed. Volcano plots demonstrating differential gene expression after radiotherapy of TC32 Ewing tumors developed in hu-CD34^+^ or NSG mice. *n* = 9 humanized mice and *n* = 6 NSG mice. **C,** Venn diagram depicting the number of significantly DEGs after radiotherapy when Ewing sarcoma tumors are developed in a hu-cD34^+^ vs. NSG model. **D,** Pathway analysis performed on the shared DEGs after radiation in immunocompetent mouse model utilizing the EnrichR bioplanet 2019 library. **E,** Upstream analysis utilizing IPA performed on shared gene lists identifies IFNγ and IRF3 as predicted upstream regulators. [**A,** Created in BioRender. Daley, J. (2025) https://BioRender.com/a3auqlx.]

We thus sought to determine the immunomodulatory effect of radiotherapy on the Ewing sarcoma TME. To do this, Ewing sarcomas were established using TC32 Ewing sarcoma cells in both the hu-CD34^+^ and NSG mouse models and were allowed to develop for 3 weeks. Tumors were then treated with 5 Gy radiotherapy as a single fraction (vs. 0 Gy control) and tumors were harvested 7 days from the start of treatment. We utilized a 5 Gy, sub-ablative dose of radiotherapy to induce TGFβ signaling in the TME (52) while still allowing for viable tumor and immune cells to persist, thus permitting the analysis of the impact of radiotherapy on Ewing sarcoma immunobiology. A second human Ewing sarcoma cell line, A673, was also evaluated (Fig. 3A). RNA isolated from flash-frozen tumors was subjected to bulk RNA-seq and the resulting data were analyzed. Transcriptional data underwent unsupervised clustering and demonstrated that transcriptional profiles of Ewing sarcomas treated with radiotherapy and developed in the hu-CD34^+^ model cluster separately from Ewing sarcoma treated with radiotherapy and developed in immunodeficient mice (Supplementary Fig. S11). When comparing the transcriptome of tumors subjected to radiotherapy versus control, we see that greater transcriptional modulation occurs after radiotherapy in Ewing sarcoma tumors developed in the hu-CD34^+^ mice as compared with tumors developed in NSG mice (Fig. 3B). A total of 2,281 significantly DEGs (*P* value < 0.05, fold change ≥1.5) were identified after radiation (as compared with no radiation) in hu-CD34^+^ tumors utilizing the TC32 cell line. Specifically, we found upregulation of genes associated with antigen presentation (*HLA-A*, *HLA-B*, and *HLA-C*) following tumor treatment with radiotherapy. In contrast, in the NSG model, we noted fewer transcriptional changes following radiotherapy, with a total of 296 significantly DEGs (*P* value < 0.05, fold change ≥1.5). We also observed greater radiotherapy-induced transcriptional changes in A673 Ewing sarcoma tumors when utilizing an immunocompetent model (Supplementary Fig. S12). Pathway analysis was performed on RNA-seq data from both TC32 and A673 tumors developed in the hu-CD34^+^ model and significant (*P* < 0.05) upregulation of pathways involved in interferon signaling, antigen presentation, and T-cell receptor–induced apoptosis was noted in both TC32 and A673 tumors (Supplementary Fig. S13). These findings clearly demonstrate the importance of an intact immune system when investigating the impact of radiotherapy on the Ewing sarcoma TME.

To further analyze radiotherapy-induced transcriptional changes in an immunocompetent model of Ewing sarcoma, we identified 22 overlapping genes that were differentially expressed following radiotherapy in tumors developed from both TC32 and A673 cell lines in hu-CD34^+^ mice. No overlapping DEGs after radiotherapy were identified in tumors developed in immunodeficient mice (Fig. 3C). We hypothesize that the limited overlap in differential gene expression between tumors developed from both cell lines is due to a difference in radiation sensitivity between the two cell lines, which we observed during *in vitro* treatment of these cell lines with radiotherapy (Supplementary Fig. S14). Pathway analysis of these overlapping genes again identified upregulation of pro-inflammatory pathways after radiotherapy, such as interferon signaling (Fig. 3D). We then assessed predicted upstream regulators of these findings utilizing IPA. Both IFNγ and IRF3 were predicted to be upstream regulators of the overlapping gene signatures induced by radiotherapy in Ewing sarcoma (Fig. 3E). Together, these data demonstrate that pro-inflammatory pathways, including interferon signaling, are upregulated following radiotherapy in Ewing sarcoma. When considering the hu-CD34^+^*in vivo* data in the context of the human Ewing sarcoma data presented in Fig. 1 (demonstrating an abundance of immunosuppressive TGFβ in the Ewing sarcoma TME), we next sought to determine the impact of TGFβ inhibition on the Ewing sarcoma TME following radiotherapy.

### The trivalent ligand trap (RER) effectively depletes TGFβ *in vivo*

**Figure 4:**
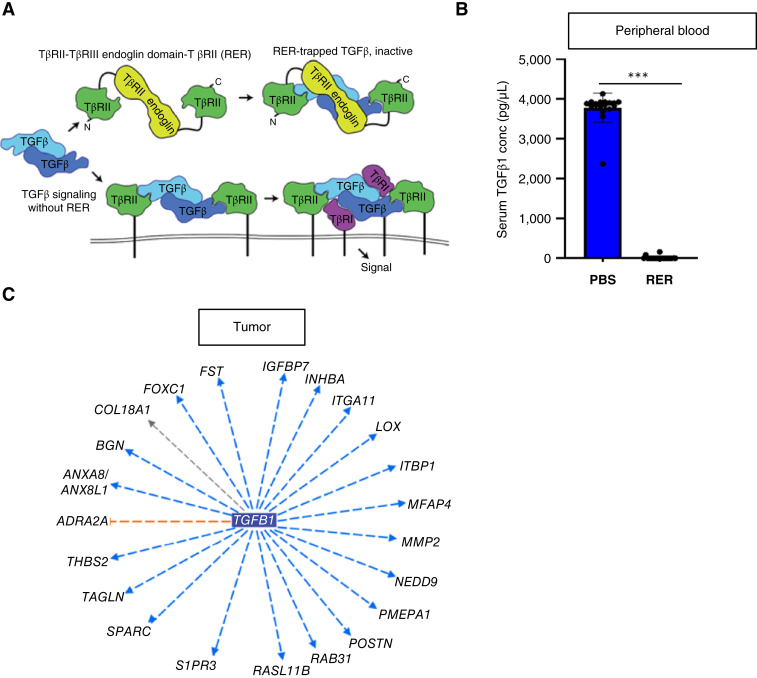
The trivalent ligand trap (RER) effectively depletes TGFβ *in vivo.***A,** Schematic of the mechanism of action of TGFβ1 ligand trap RER and interaction with TGBRII receptor. **B,** Comparison of TGFβ1 levels in the serum of mice at the time of sacrifice after receiving intraperitoneal injections of RER or PBS vehicle control. ***, *P* < 0.001. Error bars represent SD. A *t* test was performed for statistical analysis. **C,** Bulk RNA-seq was performed on tumors developed in hu-CD34^+^ mice that underwent treatment with radiotherapy on day 1 and received either RER or PBS on days 1 to 6. IPA performed on DEGs in tumors from mice receiving RER during radiotherapy demonstrates that TGFB1 is predicted to be inhibited upstream of the DEGs. Genes in the outer circle are differentially expressed; blue lines indicate the genes for which downregulation predicts TGFB1 inhibition.

To investigate the role of TGFβ inhibition in modulating the Ewing sarcoma immune microenvironment following radiotherapy, we tested the ability of RER, a trivalent ligand trap for TGFβ, to inhibit TGFβ in our hu-CD34^+^ Ewing sarcoma model. RER consists of the endoglin-like domain of the rat TGFβ co-receptor betaglycan (BGE, or E) sandwiched between the human TGFβ type II receptor extracellular domains (RII or R) on both the N- and C-terminus, as previously described (31). As illustrated, RER binds and inhibits TGFβ dimers, disabling their ability to serve as receptor ligands (Fig. 4A). Because TGFβ1, TGFβ2, and TGFβ3 all bind and participate in downstream signaling via the TGFβR2 receptor, RER is predicted to trap and inhibit the effects of all TGFβ isoforms. The safety and feasibility of TGFβ ligand traps, like RER, were recently validated in human trials (53). To assess the efficacy of RER in our model, Ewing sarcoma tumors were established in hu-CD34^+^ mice utilizing the TC32 Ewing sarcoma cell line. Mice were treated with either RER (50 mcg) or vehicle control (PBS) daily for 1 week prior to mouse sacrifice and serum collection. Analysis of mouse serum by TGFβ1 ELISA demonstrates that RER effectively depletes systemic TGFβ1 in the hu-CD34^+^ mouse model (Fig. 4B). Next, to determine whether RER treatment alters TGFβ signaling in the Ewing sarcoma TME, we examined the transcriptional profiles of Ewing sarcomas treated with radiation and RER versus vehicle control. IPA of bulk RNA-seq data identified *TGFB1* to be predicted to be significantly inhibited upstream from the DEGs identified (Fig. 4C). These data demonstrate that RER is a potent inhibitor of TGFβ in our *in vivo* model, allowing for both systemic and intra-tumoral TGFβ inhibition.

### TGFβ inhibition during radiotherapy increases immune infiltration of Ewing sarcoma tumors

**Figure 5:**
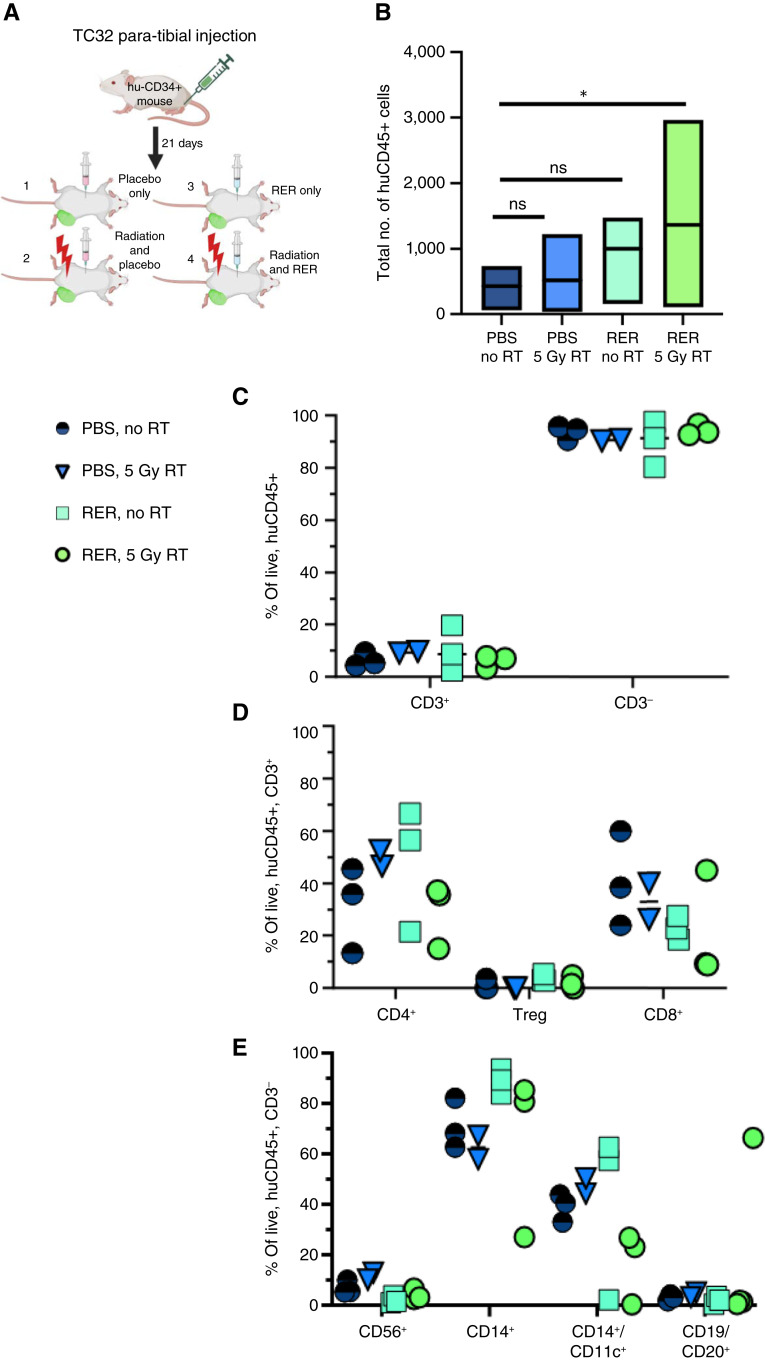
TGFβ inhibition during radiotherapy increases immune infiltration of Ewing sarcomas. **A,** Experimental schematic depicting the four treatment groups. **B,** Immunophenotyping was performed on single-cell suspensions derived from TC32 Ewing tumors established in hu-CD34^+^ mice. The total number of huCD45^+^ cells was determined in each tumor. Box plot represents average total hu-CD45+ count across three biologic replicates, line represents mean, and top and bottom limits of box are the top and bottom quartiles, respectively. *, *P* < 0.05; ns = *P* > 0.05. **C,** Immunophenotyping was performed on huCD34^+^ tumor single-cell suspensions. Samples were stained using multispectral panel (see “Materials and Methods”). Samples were analyzed on a Cytek Aurora spectral flow cytometer and then unmixed and .fcs files were imported into FlowJo for manual gating. The manual gating strategy is available in Supplementary Fig. S15. Proportion of live, huCD45^+^ cells that are CD3^+^ or CD3^−^ are shown (T cell marker). **D,** Proportion of CD3^+^ cells that are CD4^+^ T cells, regulatory T cells (Treg), or CD8^+^ cells are shown. **E,** Proportion of CD3^−^ cells that are CD56^+^ (NK cell marker), CD14^+^ (monocyte marker), CD14^+^/CD11c^+^ (myeloid derived suppressor cell), and CD19/CD20^+^ (B cell marker) are shown. RT, radiotherapy. [**A,** Created in BioRender. Daley, J. (2025) https://BioRender.com/jn2ii5x]

After identifying an effective method to inhibit TGFβ *in vivo* (RER), we next focused on understanding the functional impact of TGFβ inhibition during radiotherapy on Ewing sarcoma tumors in our hu-CD34^+^ model. We first investigated the effect of TGFβ inhibition during radiotherapy on immune infiltration into Ewing sarcoma tumors. Ewing sarcoma tumors were established in the hu-CD34^+^ model and mice were treated in one of four groups: (i) vehicle control and no radiotherapy, (ii) vehicle control and radiotherapy, (iii) RER and no radiotherapy, and (iv) RER and radiotherapy (Fig. 5A). At the time of sacrifice, tumors were harvested, processed into single-cell suspensions, and immunophenotyped by flow cytometry. Analysis of the human CD45^+^ leukocytes infiltrating the Ewing sarcoma tumors revealed that tumors from mice treated with RER in combination with radiotherapy demonstrate a significant (*P* < 0.05) increase in tumor-infiltrating leukocytes as compared with untreated Ewing sarcomas (Fig. 5B). Neither radiation alone nor RER alone resulted in a significant different in tumor-infiltrating leukocytes. Analysis of immune cell sub-populations demonstrates no significant difference in the ratio of CD3^+^ T cell and CD3^−^ (non-T cell) populations between each experimental group (Fig. 5C). In the CD3^+^ T-cell population, we also demonstrate no significant difference in CD4+ or CD8^+^ T-cell populations between treatment groups (Fig. 5D). We further determine that in all tumors analyzed, the predominant immune cell population is CD3^−^, with a predominant myeloid population that is CD14^+^ and CD14^+^CD11c^+^ (Fig. 5E; Supplementary Fig. S15). Our finding that TGFβ inhibition during radiotherapy significantly increases the total number of hu-CD34^+^ cells infiltrating Ewing sarcomas suggests that the immune microenvironment of Ewing sarcoma is modifiable and that combining TGFβ inhibition with radiotherapy could be utilized as a tool to enhance total immune cell infiltration in human Ewing sarcomas.

### TGFβ inhibition during radiotherapy suppresses Ewing sarcoma metastasis

**Figure 6:**
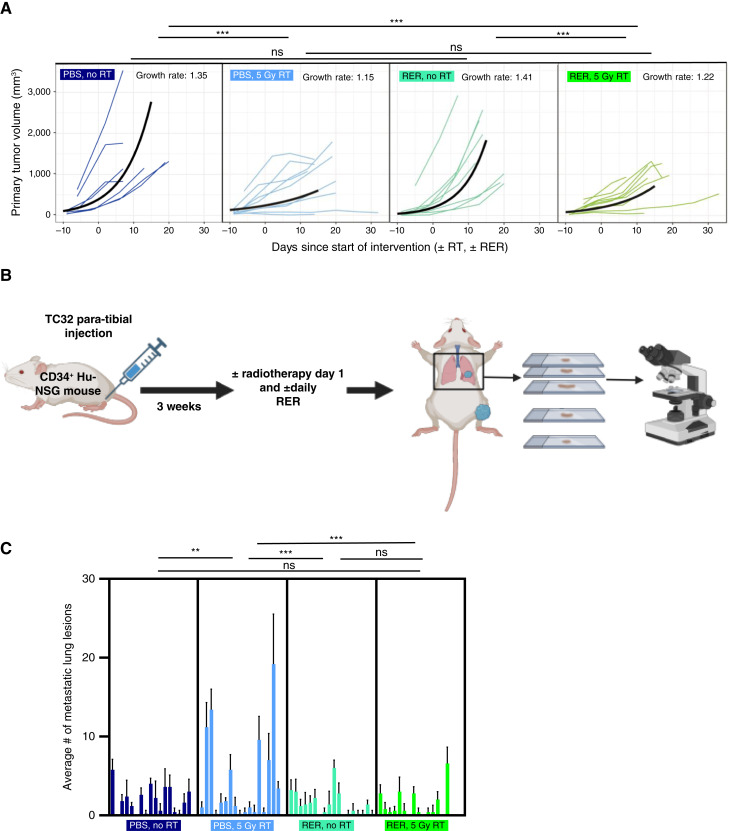
TGFβ inhibition during radiation suppresses the Ewing sarcoma metastatic phenotype. **A,** Tumors were established utilizing the TC32 cell line hu-CD34^+^ mice. 3 weeks following tumor injection, mice were treated with ± radiotherapy (5 Gy dose × 1) and ± TGFβ inhibition with daily RER injection (or PBS control). CT imaging was obtained starting 1 week following tumor cell injection and then once weekly thereafter. Tumors were allowed to develop until mice met criteria for euthanasia. Tumor volumes were determined utilizing CT imaging (see methods). Growth rate represents the average percent increase in tumor volume per day. Each colored line represents an individual mouse and the black line represents the average growth rate. Day –10 indicates 10 days prior to the start of treatment interventions. Tumor growth curves were estimated by a linear mixed model with a fixed effect for treatment and random effect for mouse. (*n* = 7 control group, *n* = 8 radiotherapy only group, *n* = 9 RER only group, and *n* = 9 radiotherapy + RER group). **B,** Schematic of the experimental design for serial sectioning of bilateral lung tissue for the analysis of metastatic disease burden. **C,** Bilateral lungs were harvested from mice in the experiment from Fig. 5 (*n* = 9 mice per group) and mice from **A** (*n* = 8 control group, *n* = 8 radiotherapy only group, *n* = 9 RER only group, *n* = 7 radiotherapy + RER group). Lung tissue was formalin fixed and paraffin embedded and underwent serial sectioning prior to review by an independent pathologist (see “Materials and Methods”). The number of metastatic foci per serial section was determined. Each bar represents the average number of metastatic lesions across serial section in individual mouse, and error bars represent SD. ***, *P* < 0.001; **, *P* < 0.01; ns = *P* value ≥ 0.05. RT, radiotherapy. [**B,** Created in BioRender. Daley, J. (2025) https://BioRender.com/cnw56f3.]

Beyond immunosuppression, TGFβ is also reported to promote tumor growth and/or metastasis in some cancer types (34). It has been shown that subpopulations of Ewing sarcoma cells termed “EWS::FLI1 low” represent an aggressive population of Ewing cells that demonstrate increased TGFβ signaling (34). Although EWS::FLI1 downregulates TGFβR2, one of the two essential TGFβ signaling receptors, these “EWS-FLI1-low” cells maintain the expression of TGFBR2 and participate in TGFβ signaling (54, 55). With these previous findings in mind, we sought to determine the effect of TGFβ inhibition during radiotherapy on primary Ewing sarcoma tumor growth and metastatic potential. To evaluate the impact of TGFβ inhibition during radiotherapy on Ewing sarcoma primary tumor growth, Ewing sarcoma tumors were established in our hu-CD34^+^ mouse model and mice were randomized into the same four groups shown in Fig. 5 (± single fraction of radiotherapy and ± RER). Tumor volume was determined utilizing microCT imaging and demonstrated that both radiotherapy and radiotherapy in combination with RER significantly decreased tumor growth rates when compared with no radiation or with RER alone (Fig. 6A). Combining RER with radiotherapy did not significantly affect primary tumor growth when compared with radiotherapy alone (Fig. 6A; Supplementary Fig. S16 for survival). RT-PCR of RNA extracted from primary tumors demonstrated no significant net difference in EWSR1::FLI1 expression across treatment groups (Supplementary Fig. S17).

Given previous reports of increased TGFβ signaling in Ewing sarcoma cell populations with a pro-metastatic phenotype *in vitro* (34), we next compared rates of metastasis with ± TGFβ inhibition by examining lung tissue for the presence of metastases in the mice utilized in the experiments shown in both Figs. 5 and 6. Bilateral lungs were isolated at the time of sacrifice and tissue was formalin-fixed and paraffin-embedded. The resulting FFPE blocks underwent serial sectioning and lung tissue underwent review for the presence of metastases by an independent pathologist blinded to treatment conditions (Fig. 6B; Supplementary Fig. S18). Quantification of these data revealed a significant increase in pulmonary metastases in our humanized model when tumors were treated with a single fraction of radiotherapy alone when compared with no radiation or RER alone (Fig. 6C). Notably, when mice are treated with RER during radiotherapy, this effect of radiotherapy in enhancing metastasis is significantly (*P* < 0.05) abrogated, suggesting a role for TGFβ in promoting Ewing sarcoma lung metastases in the setting of radiotherapy.

Next, to investigate potential mechanisms by which TGFβ inhibition reduces Ewing sarcoma metastases following radiotherapy in our model, we analyzed the transcriptional profile of these tumors. Pathway analysis was performed on transcripts significantly downregulated in tumors that received radiotherapy and TGFβ inhibition, compared with tumors receiving radiotherapy alone, and it revealed that three of the top 10 downregulated pathways involved TGFβ signaling as anticipated (Supplementary Fig. S19). Additionally, significant downregulation of pathways involved in ECM organization and interactions (integrins) was noted. Taken together, these data demonstrate that although combining TGFβ inhibition with sub-ablative dosing of radiotherapy does not significantly affect primary tumor growth, TGFβ inhibition significantly reduces tumor metastatic potential during radiotherapy, and downregulation of pro-metastatic ECM genes may play a role in this effect of TGFβ.

## Discussion

Here, we demonstrate, using scRNA-seq analysis, that immune cells express *TGFB1* in human Ewing tumors. We then utilize an *in vivo*, immunocompetent humanized (hu-CD34^+^) mouse model to demonstrate that TGFβ inhibition enhances total immune cell infiltration and reduces lung metastases following radiotherapy in Ewing sarcoma. The major implications of our study include that (i) we have developed an immunocompetent mouse model tool for the study of the Ewing sarcoma TME and the preclinical evaluation of TME-modulating agents in Ewing sarcoma, (ii) our data support the potential use of TGFβ inhibition during radiotherapy to promote immune cell infiltration into Ewing tumors, and (iii) our data indicate that TGFβ inhibition may reduce metastatic potential following radiotherapy in Ewing sarcoma. We will now discuss each of these points in greater detail.

First, the identification of appropriate preclinical models that can accurately predict the success of new therapies in early-phase clinical trials remains a challenge (56, 57). Importantly, the role of the immune component of the TME in driving tumor response to therapy, and potential resistant mechanisms, is increasingly recognized as a crucial consideration in the design of new therapeutic strategies (7). There are no robust syngeneic or transgenic mouse models of Ewing sarcoma (17), so for this study, we developed a humanized mouse model of Ewing sarcoma utilizing a hu-CD34^+^ model to be able to ask specific questions about how radiotherapy and TGFβ inhibition affect the tumor immune response. Baseline Ewing sarcoma tumor infiltration with immune cells in our hu-CD34^+^ model is low, similar to the low level of immune infiltrate noted in primary human Ewing sarcoma tumors, including our own prior work (22). Our data clearly demonstrate transcriptional signature differences between identical Ewing sarcoma developed in hu-CD34^+^ versus NSG mice, thus highlighting the influence of immune cells on Ewing sarcoma tumor biology. Utilization of this model will now allow our Ewing sarcoma research community to perform studies addressing specific immune-biological questions and critically important preclinical testing of agents that may have potential TME or immunomodulatory effects. Incorporation of this model into preclinical testing of Ewing sarcoma holds the potential to elucidate immune-driven mechanisms of therapeutic resistance or efficacy.

Second, utilizing our hu-CD34^+^ orthotopic mouse model of Ewing sarcoma, we demonstrate that combining TGFβ inhibition with radiotherapy results in increased total immune cell (CD45^+^) infiltration into Ewing sarcomas in comparison with no treatment. When considering clinical applications of this finding, cellular therapies are an important consideration. A key barrier to the efficacy of cellular therapies for solid tumors, including Ewing sarcoma, is the limited infiltration of engineered cell therapies into the tumors (58–60). Our data suggest that priming tumors with TGFβ inhibition and radiotherapy prior to the delivery of cellular therapies may improve their infiltration into the tumor. Testing this concept is an important future direction of this work.

Third, very little is currently understood about the mechanisms regulating Ewing sarcoma lung metastases (61). Our current findings demonstrate that more spontaneous lung metastases develop at baseline when comparing orthotopic Ewing sarcoma tumors developed in hu-CD34^+^ mice versus NSG mice, suggesting that immune mechanisms contribute to Ewing sarcoma metastasis and that there may an advantage to utilizing our hu-CD34^+^ model in future studies specifically focused on Ewing sarcoma lung metastases. We also demonstrate that a single fraction of radiotherapy leads to an increase in Ewing sarcoma lung metastases in humanized mice. Although the exact mechanism of this effect of radiotherapy is unknown, we postulate that this dose of sub-ablative radiotherapy could induce enhanced leakiness of the tumor vasculature (62) or bleeding into the tumor that helps assist tumor cell escape. Importantly, we find that TGFβ inhibition abrogates radiation-induced lung metastases and it is interesting to speculate on the potential clinical implications of this finding. It is important to note that the dose of radiotherapy utilized for the treatment of metastatic and relapsed Ewing sarcoma in our patients is significantly higher than the doses used in the experiments reported here using our humanized mouse model (27, 28, 51). There have been recent reports in the literature indicating that radiotherapy can enhance metastasis via induction of increased populations of myeloid-derived suppressor cells (63), which may be relevant to our findings with regard to TGFβ inhibition decreasing metastases following radiotherapy in our model. Historically, the effect of TGFβ signaling in Ewing sarcomas has been assumed to be of limited consequence because of the repression of TGFβR2 by the EWS::FLI1 oncoprotein (64). However, in the current study, our data highlight the importance of TGFβ biology in Ewing sarcoma. Future preclinical studies will test additional TGF-β inhibitors in combination with radiotherapy with the goal of identifying promising new treatment approaches to reduce subsequent lung relapses.

## Supplementary Material

Figure S1 Figure S1. TGFβ1 expression is present in the immune cell compartment of human Ewing tumors.

Figure S2 Figure S2. TGFβ1 is the predominant TGFβ isoform expressed by immune cells in human Ewing tumors.

Figure S3 Figure S3. Reconstitution with huCD45+ cells is verified prior to tumor implantation.

Figure S4 Figure S4. CT imaging of mouse tumors demonstrates bony destruction when tumors are established in para-tibial location.

Figure S5 Figure S5. Negative control tissue for CD99 and NKX2.2 staining.

Figure S6 Figure S6. Human natural killer (NK) cells are present in the peripheral blood of humanized mice.

Figure S7 Figure S7. Bulk RNA sequencing was performed on TC32 Ewing sarcoma tumors developed in either hu-CD34+ or NSG mouse models.

Figure S8 Figure S8. Ewing sarcoma tumors developed in hu-CD34+ mice demonstrate upregulation of ECM pathways.

Figure S9 Figure S9. TGFβ1 levels are increased in an immunocompetent mouse model of Ewing sarcoma.

Figure S10 Figure S10. Ewing sarcoma tumor growth is similar in humanized and NSG mouse models.

Figure S11 Figure S11. Distinct tumor transcriptional signatures following radiation therapy are noted in Ewing sarcomas developed in hu-cD34+ versus NSG mouse models.

Figure S12 Figure S12. Transcriptional modulation induced following radiation in A673 Ewing sarcoma tumors developed in hu-CD34+ versus NSG mice.

Figure S13 Figure S13. Radiation therapy induces upregulation of inflammatory pathways in Ewing sarcoma tumors developed in a hu-CD34+ mouse model.

Figure S14 Figure S14. The TC32 EwS cell line demonstrates increased sensitivity to radiation therapy compared to the A673 cell line in vitro.

Figure S15 Figure S15. Flow cytometry gating strategy.

Figure S16 Figure S16. Survival of mice treated with ± RER and ± radiation therapy.

Figure S17 Figure S17. EWS::FLI1 expression in tumors established in hu-mice.

Figure S18 Figure S18. Representative H&E images of mouse lungs used for metastatic quantification.

Figure S19 Figure S19. TGFβ signaling is decreased in tumors of mice that receive RER following radiation therapy as compared to mice that are treated with radiation therapy (RT) alone.

Table S1 Gene lists from comparisons

Table S2 Sample characteristics

Table S3 Cell counts

Table S4 immune cell infiltrates in human tumors
