# Supplementary material for: TGFβ Inhibition during Radiotherapy Enhances Immune Cell Infiltration and Decreases Metastases in Ewing Sarcoma
Source: Cancer Res Commun. 2025 Aug 27;5(8):1441–57. doi: 10.1158/2767-9764.CRC-24-0346 (PMC12380665; doi:10.1158/2767-9764.CRC-24-0346)
Supplement: Figure S1 — TGFβ1 expression is present in the immune cell compartment of human Ewing tumors. [file crc-24-0346_figure_s1_suppsf1.pptx]

## Slide 1
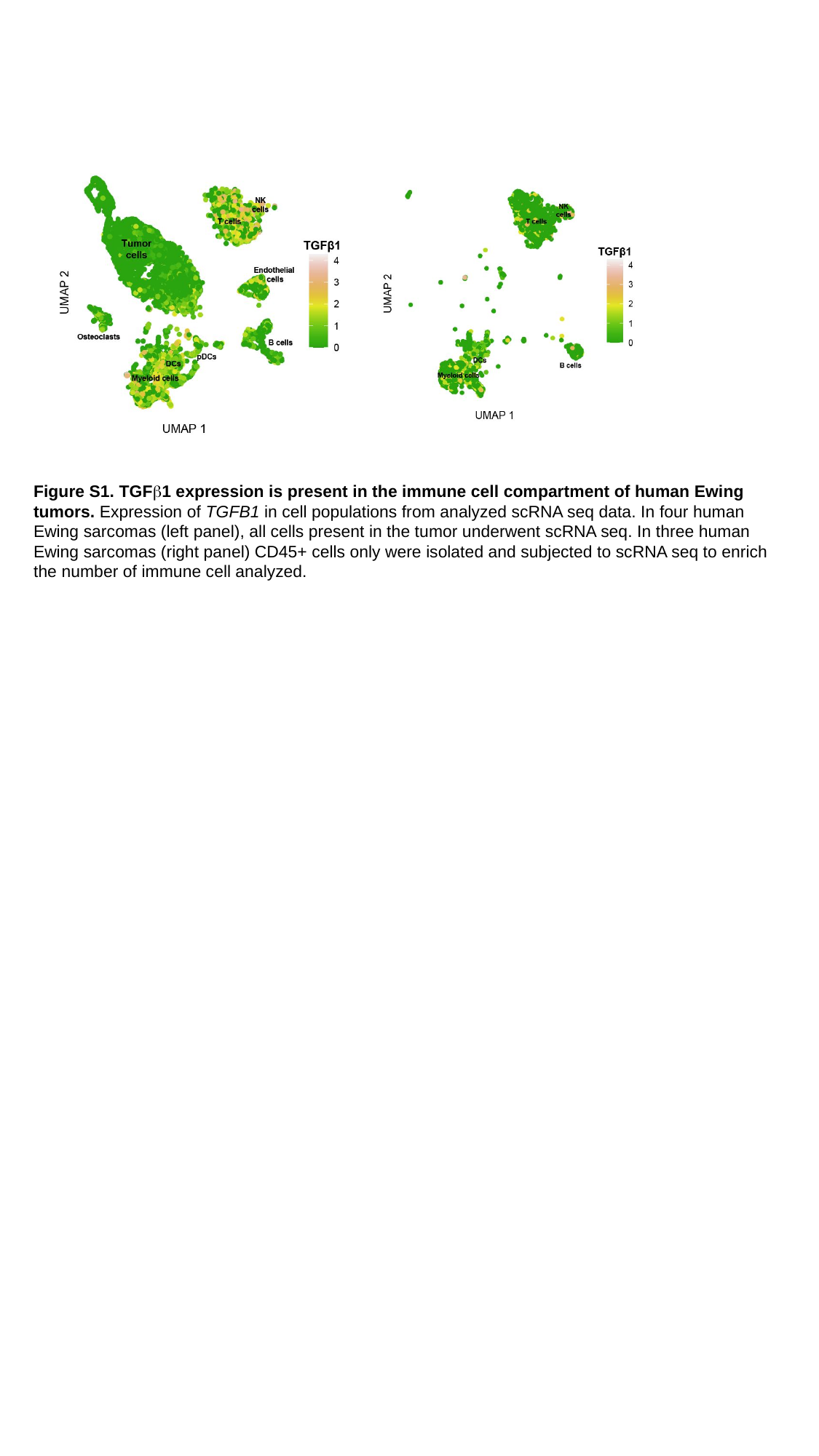

Figure S1. TGFβ1 expression is present in the immune cell compartment of human Ewing tumors. Expression of TGFB1 in cell populations from analyzed scRNA seq data. In four human Ewing sarcomas (left panel), all cells present in the tumor underwent scRNA seq. In three human Ewing sarcomas (right panel) CD45+ cells only were isolated and subjected to scRNA seq to enrich the number of immune cell analyzed.
