# Supplementary material for: TGFβ Inhibition during Radiotherapy Enhances Immune Cell Infiltration and Decreases Metastases in Ewing Sarcoma
Source: Cancer Res Commun. 2025 Aug 27;5(8):1441–57. doi: 10.1158/2767-9764.CRC-24-0346 (PMC12380665; doi:10.1158/2767-9764.CRC-24-0346)
Supplement: Figure S2 — TGFβ1 is the predominant TGFβ isoform expressed by immune cells in human Ewing tumors. [file crc-24-0346_figure_s2_suppsf2.pptx]

## Slide 1
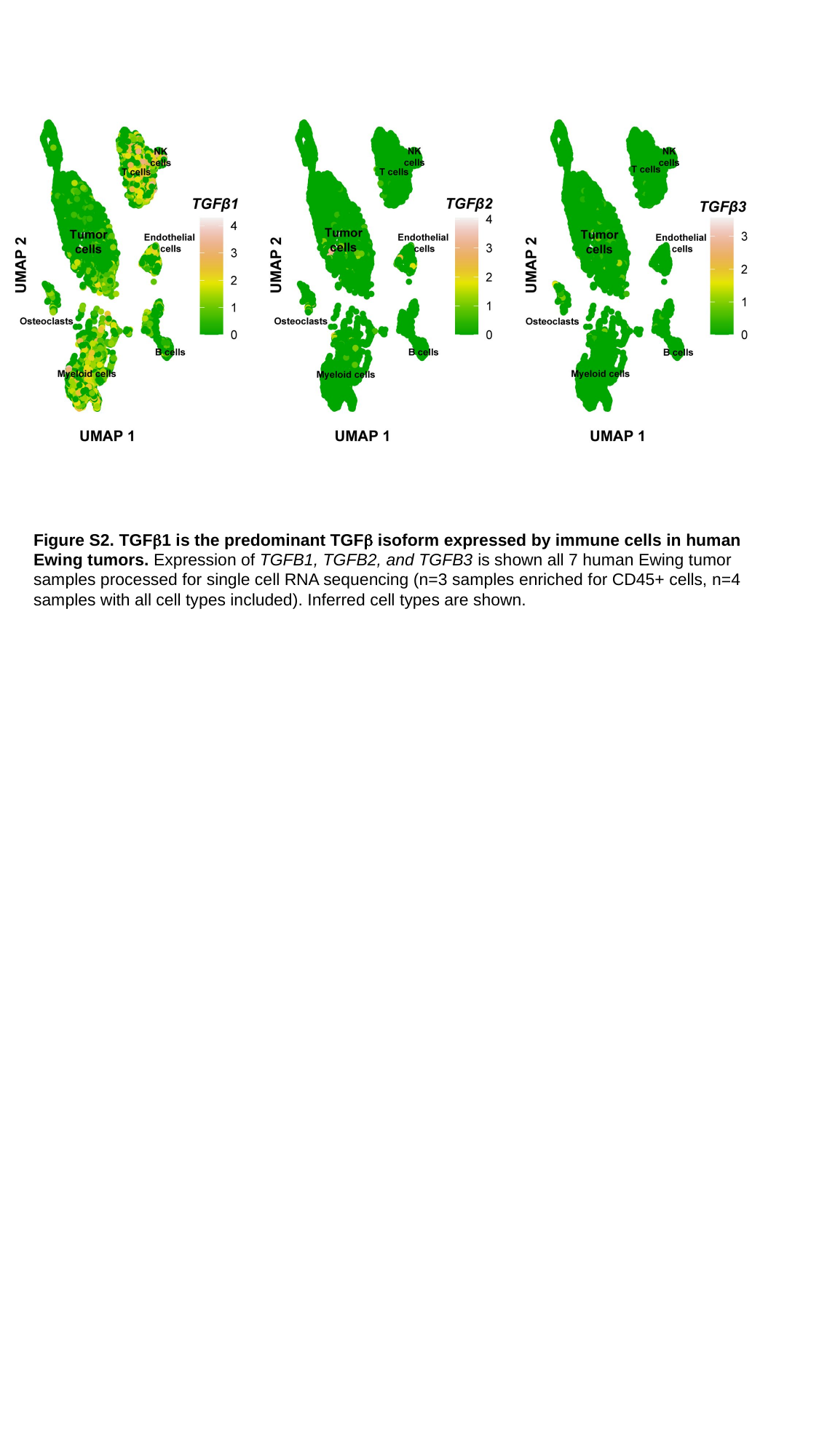

Figure S2. TGFβ1 is the predominant TGFβ isoform expressed by immune cells in human Ewing tumors. Expression of TGFB1, TGFB2, and TGFB3 is shown all 7 human Ewing tumor samples processed for single cell RNA sequencing (n=3 samples enriched for CD45+ cells, n=4 samples with all cell types included). Inferred cell types are shown.
