# Supplementary material for: TGFβ Inhibition during Radiotherapy Enhances Immune Cell Infiltration and Decreases Metastases in Ewing Sarcoma
Source: Cancer Res Commun. 2025 Aug 27;5(8):1441–57. doi: 10.1158/2767-9764.CRC-24-0346 (PMC12380665; doi:10.1158/2767-9764.CRC-24-0346)
Supplement: Figure S3 — Reconstitution with huCD45+ cells is verified prior to tumor implantation. [file crc-24-0346_figure_s3_suppsf3.pptx]

## Slide 1
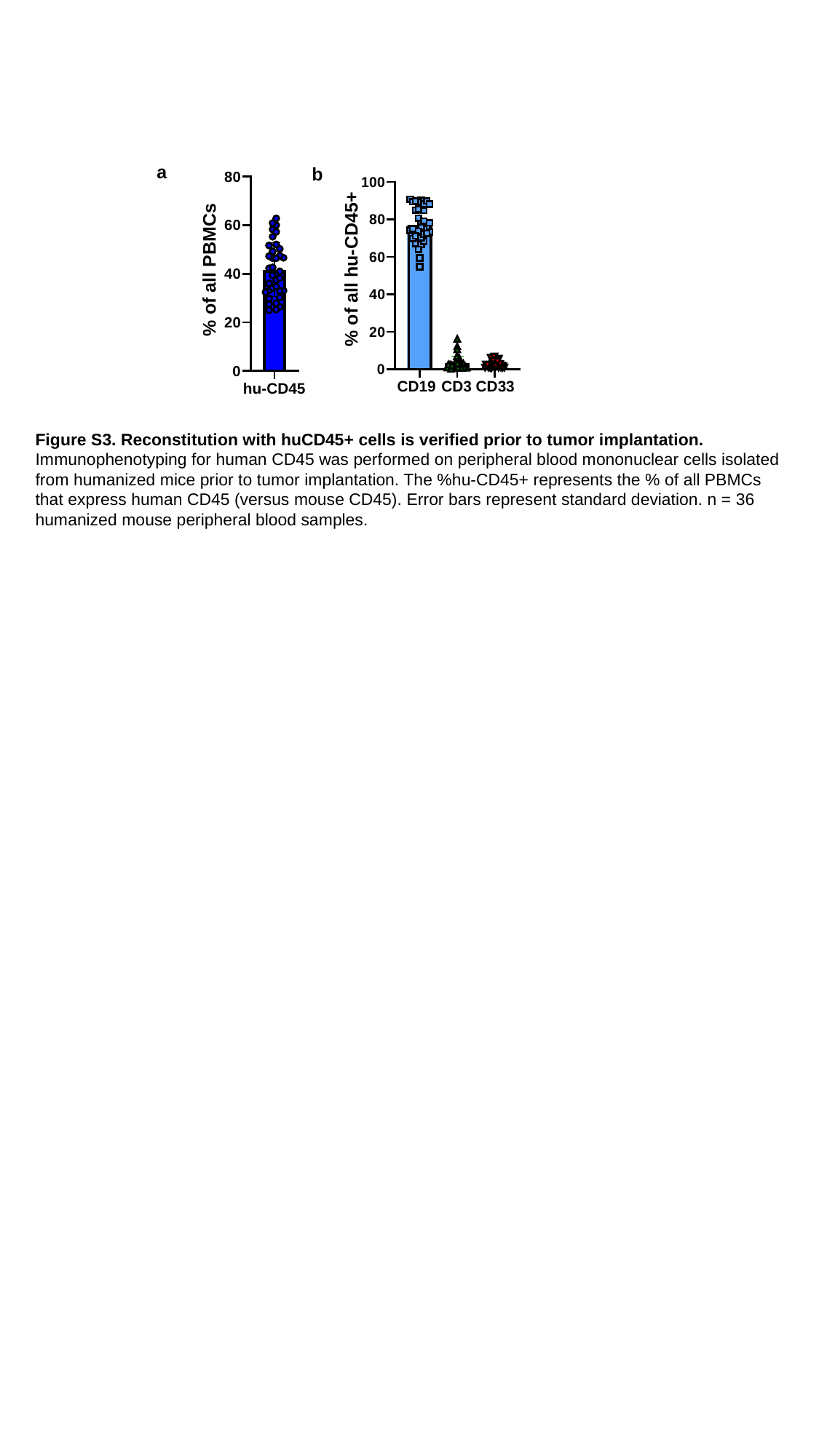

a
b
% of all hu-CD45+
% of all PBMCs
CD19
CD3
CD33
hu-CD45
Figure S3. Reconstitution with huCD45+ cells is verified prior to tumor implantation. Immunophenotyping for human CD45 was performed on peripheral blood mononuclear cells isolated from humanized mice prior to tumor implantation. The %hu-CD45+ represents the % of all PBMCs that express human CD45 (versus mouse CD45). Error bars represent standard deviation. n = 36 humanized mouse peripheral blood samples.
