# Supplementary material for: TGFβ Inhibition during Radiotherapy Enhances Immune Cell Infiltration and Decreases Metastases in Ewing Sarcoma
Source: Cancer Res Commun. 2025 Aug 27;5(8):1441–57. doi: 10.1158/2767-9764.CRC-24-0346 (PMC12380665; doi:10.1158/2767-9764.CRC-24-0346)
Supplement: Figure S4 — CT imaging of mouse tumors demonstrates bony destruction when tumors are established in para-tibial location. [file crc-24-0346_figure_s4_suppsf4.pptx]

## Slide 1
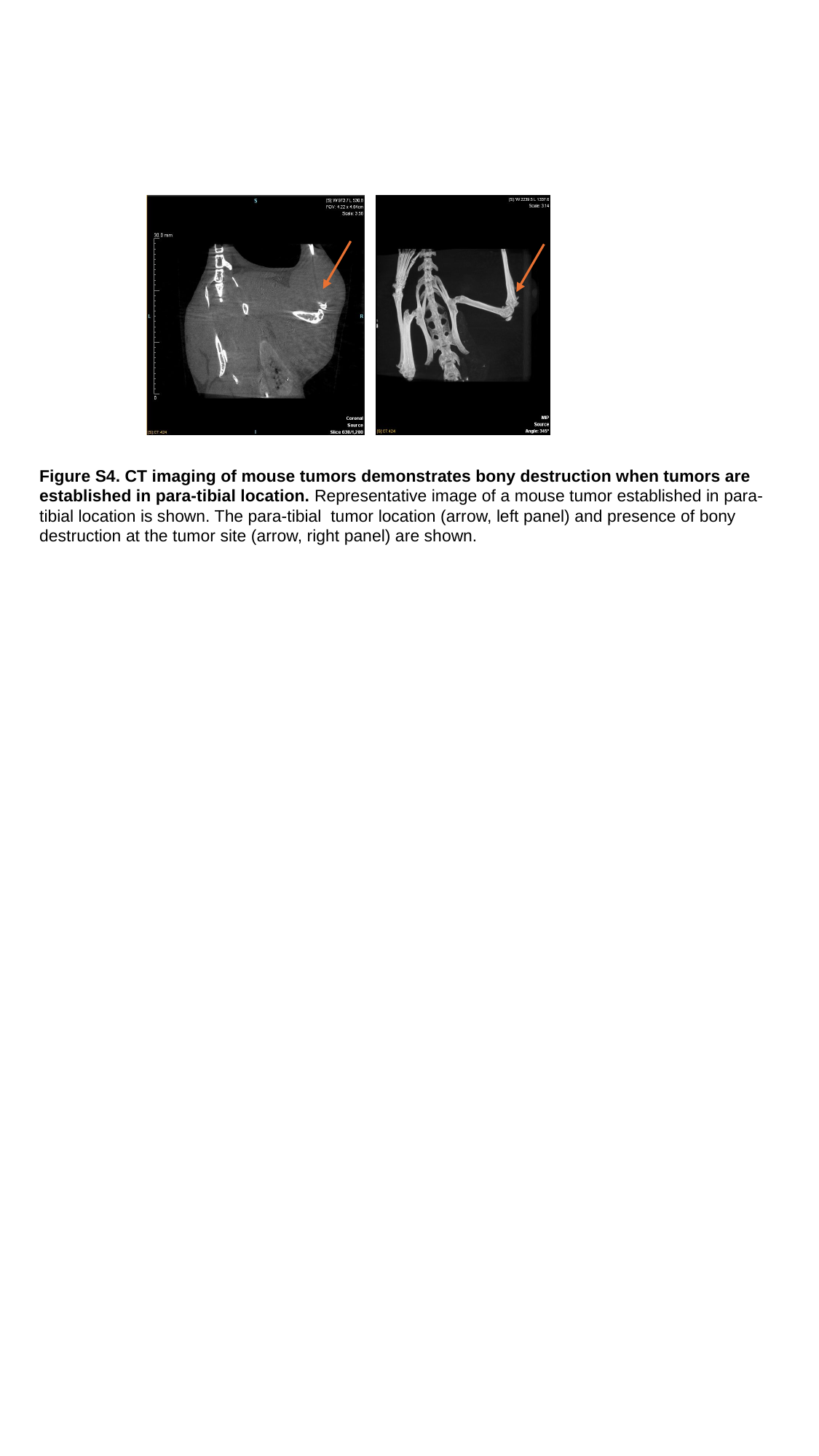

Figure S4. CT imaging of mouse tumors demonstrates bony destruction when tumors are established in para-tibial location. Representative image of a mouse tumor established in para-tibial location is shown. The para-tibial tumor location (arrow, left panel) and presence of bony destruction at the tumor site (arrow, right panel) are shown.
