# Supplementary material for: TGFβ Inhibition during Radiotherapy Enhances Immune Cell Infiltration and Decreases Metastases in Ewing Sarcoma
Source: Cancer Res Commun. 2025 Aug 27;5(8):1441–57. doi: 10.1158/2767-9764.CRC-24-0346 (PMC12380665; doi:10.1158/2767-9764.CRC-24-0346)
Supplement: Figure S5 — Negative control tissue for CD99 and NKX2.2 staining. [file crc-24-0346_figure_s5_suppsf5.pptx]

## Slide 1
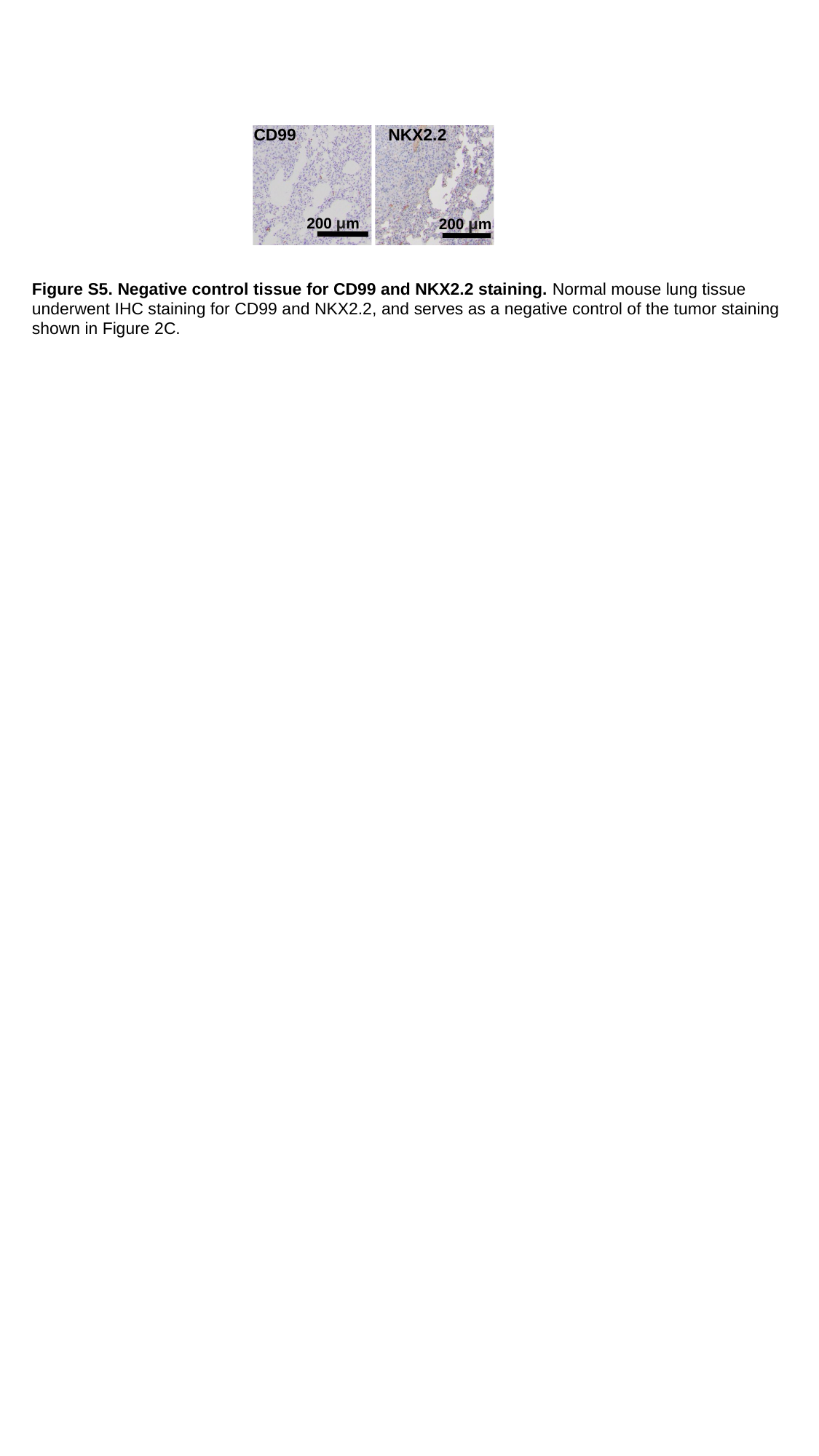

CD99
NKX2.2
200 µm
200 µm
Figure S5. Negative control tissue for CD99 and NKX2.2 staining. Normal mouse lung tissue underwent IHC staining for CD99 and NKX2.2, and serves as a negative control of the tumor staining shown in Figure 2C.
