# Supplementary material for: TGFβ Inhibition during Radiotherapy Enhances Immune Cell Infiltration and Decreases Metastases in Ewing Sarcoma
Source: Cancer Res Commun. 2025 Aug 27;5(8):1441–57. doi: 10.1158/2767-9764.CRC-24-0346 (PMC12380665; doi:10.1158/2767-9764.CRC-24-0346)
Supplement: Figure S6 — Human natural killer (NK) cells are present in the peripheral blood of humanized mice. [file crc-24-0346_figure_s6_suppsf6.pptx]

## Slide 1
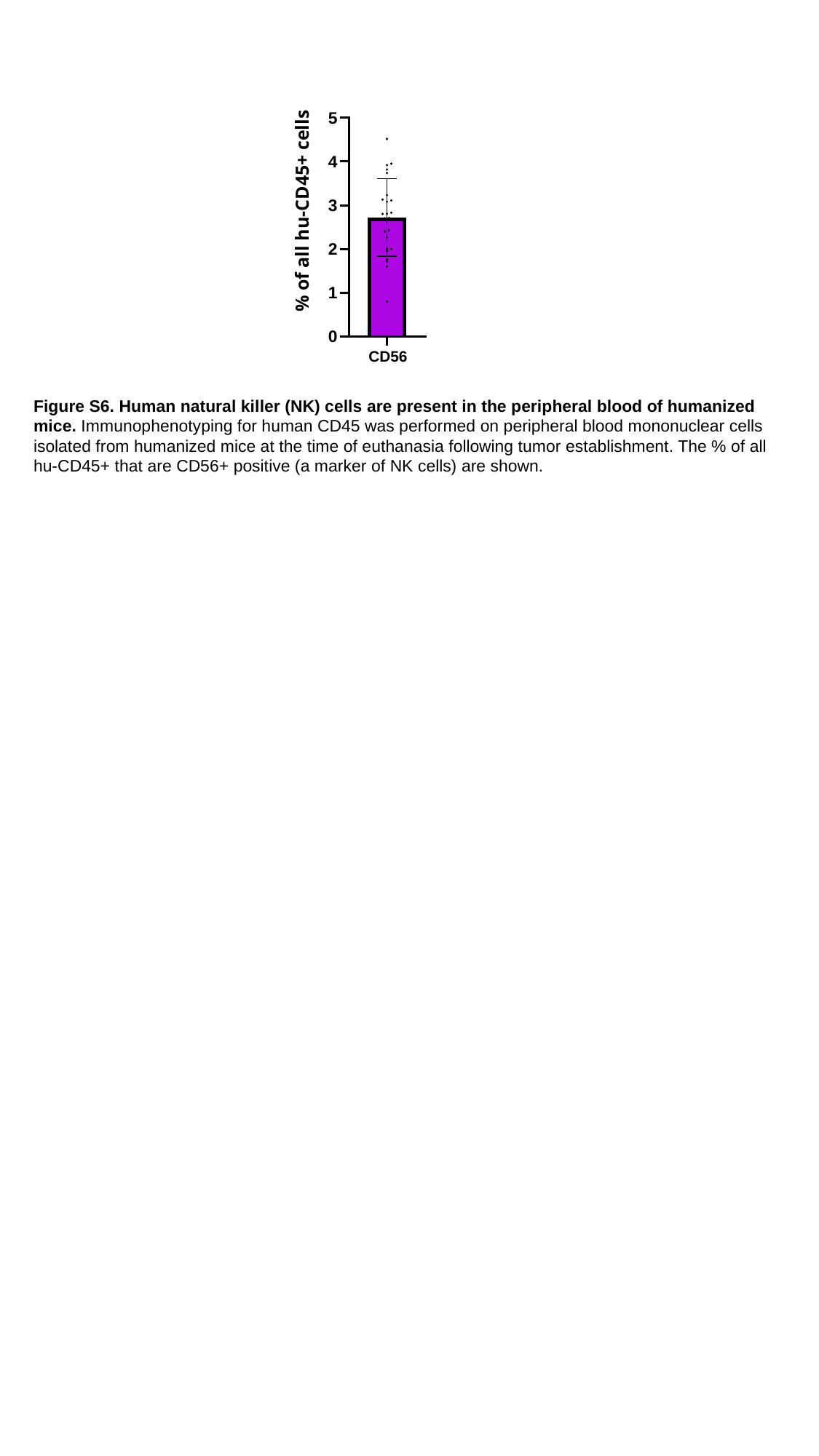

% of all hu-CD45+ cells
CD56
Figure S6. Human natural killer (NK) cells are present in the peripheral blood of humanized mice. Immunophenotyping for human CD45 was performed on peripheral blood mononuclear cells isolated from humanized mice at the time of euthanasia following tumor establishment. The % of all hu-CD45+ that are CD56+ positive (a marker of NK cells) are shown.
