# Supplementary material for: TGFβ Inhibition during Radiotherapy Enhances Immune Cell Infiltration and Decreases Metastases in Ewing Sarcoma
Source: Cancer Res Commun. 2025 Aug 27;5(8):1441–57. doi: 10.1158/2767-9764.CRC-24-0346 (PMC12380665; doi:10.1158/2767-9764.CRC-24-0346)
Supplement: Figure S7 — Bulk RNA sequencing was performed on TC32 Ewing sarcoma tumors developed in either hu-CD34+ or NSG mouse models. [file crc-24-0346_figure_s7_suppsf7.pptx]

## Slide 1
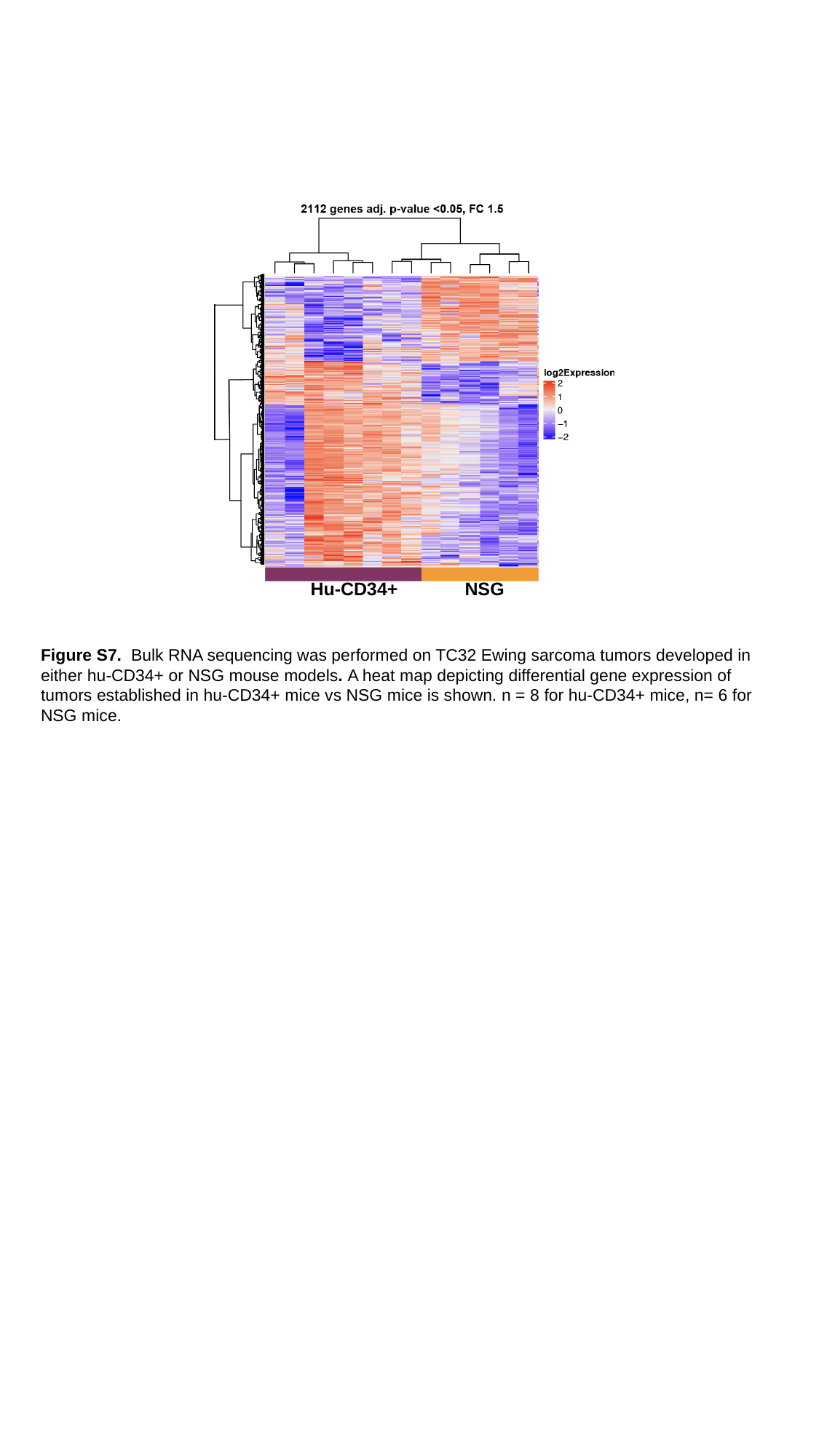

Hu-CD34+
NSG
Figure S7. Bulk RNA sequencing was performed on TC32 Ewing sarcoma tumors developed in either hu-CD34+ or NSG mouse models. A heat map depicting differential gene expression of tumors established in hu-CD34+ mice vs NSG mice is shown. n = 8 for hu-CD34+ mice, n= 6 for NSG mice.
