# Supplementary material for: TGFβ Inhibition during Radiotherapy Enhances Immune Cell Infiltration and Decreases Metastases in Ewing Sarcoma
Source: Cancer Res Commun. 2025 Aug 27;5(8):1441–57. doi: 10.1158/2767-9764.CRC-24-0346 (PMC12380665; doi:10.1158/2767-9764.CRC-24-0346)
Supplement: Figure S8 — Ewing sarcoma tumors developed in hu-CD34+ mice demonstrate upregulation of ECM pathways. [file crc-24-0346_figure_s8_suppsf8.pptx]

## Slide 1
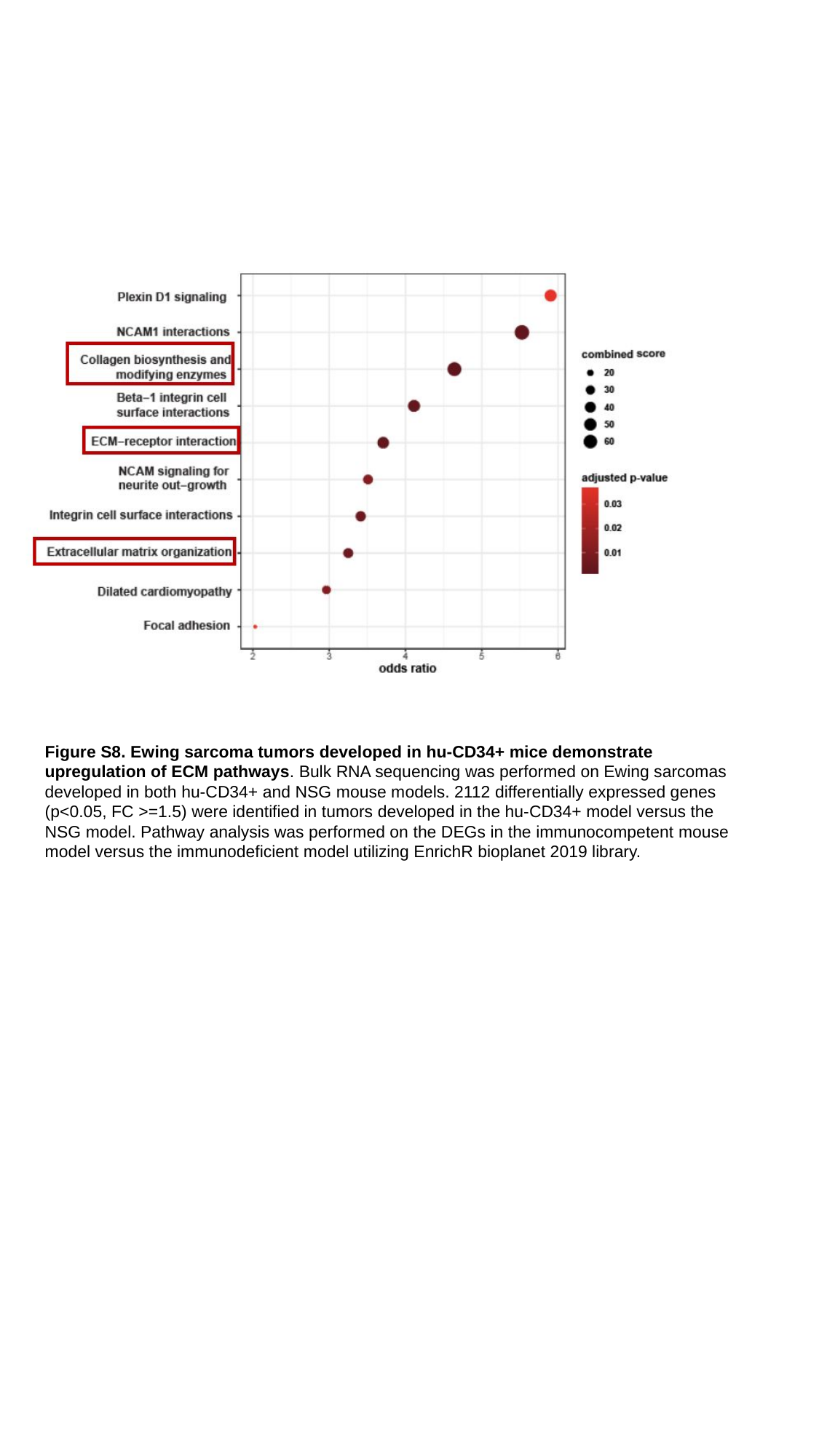

Figure S8. Ewing sarcoma tumors developed in hu-CD34+ mice demonstrate upregulation of ECM pathways. Bulk RNA sequencing was performed on Ewing sarcomas developed in both hu-CD34+ and NSG mouse models. 2112 differentially expressed genes (p<0.05, FC >=1.5) were identified in tumors developed in the hu-CD34+ model versus the NSG model. Pathway analysis was performed on the DEGs in the immunocompetent mouse model versus the immunodeficient model utilizing EnrichR bioplanet 2019 library.
