# Supplementary material for: TGFβ Inhibition during Radiotherapy Enhances Immune Cell Infiltration and Decreases Metastases in Ewing Sarcoma
Source: Cancer Res Commun. 2025 Aug 27;5(8):1441–57. doi: 10.1158/2767-9764.CRC-24-0346 (PMC12380665; doi:10.1158/2767-9764.CRC-24-0346)
Supplement: Figure S9 — TGFβ1 levels are increased in an immunocompetent mouse model of Ewing sarcoma. [file crc-24-0346_figure_s9_suppsf9.pptx]

## Slide 1
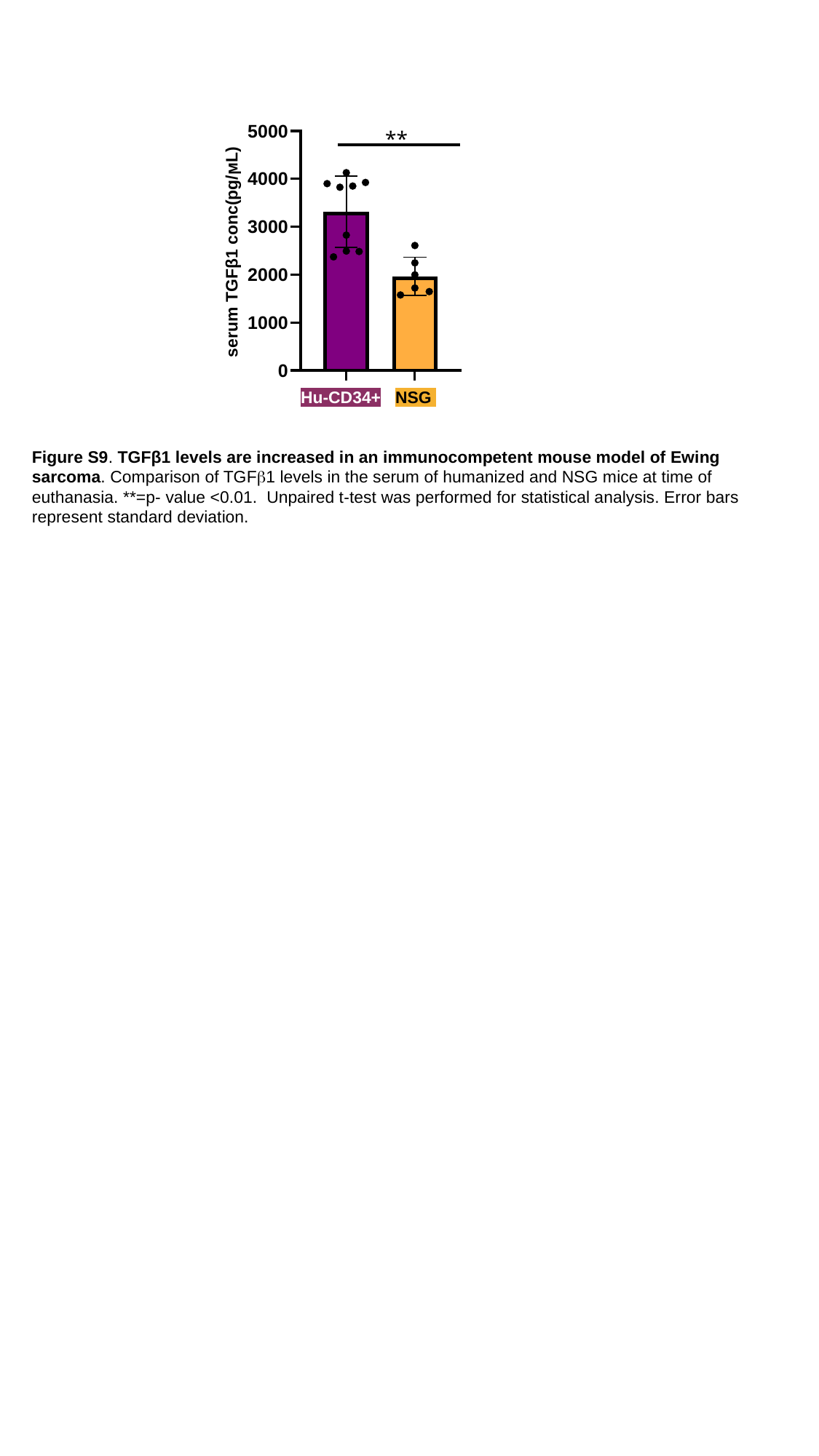

**
serum TGFβ1 conc(pg/ϻL)
Hu-CD34+
NSG
Figure S9. TGFβ1 levels are increased in an immunocompetent mouse model of Ewing sarcoma. Comparison of TGFβ1 levels in the serum of humanized and NSG mice at time of euthanasia. **=p- value <0.01. Unpaired t-test was performed for statistical analysis. Error bars represent standard deviation.
