# Supplementary material for: TGFβ Inhibition during Radiotherapy Enhances Immune Cell Infiltration and Decreases Metastases in Ewing Sarcoma
Source: Cancer Res Commun. 2025 Aug 27;5(8):1441–57. doi: 10.1158/2767-9764.CRC-24-0346 (PMC12380665; doi:10.1158/2767-9764.CRC-24-0346)
Supplement: Figure S10 — Ewing sarcoma tumor growth is similar in humanized and NSG mouse models. [file crc-24-0346_figure_s10_suppsf10.pptx]

## Slide 1
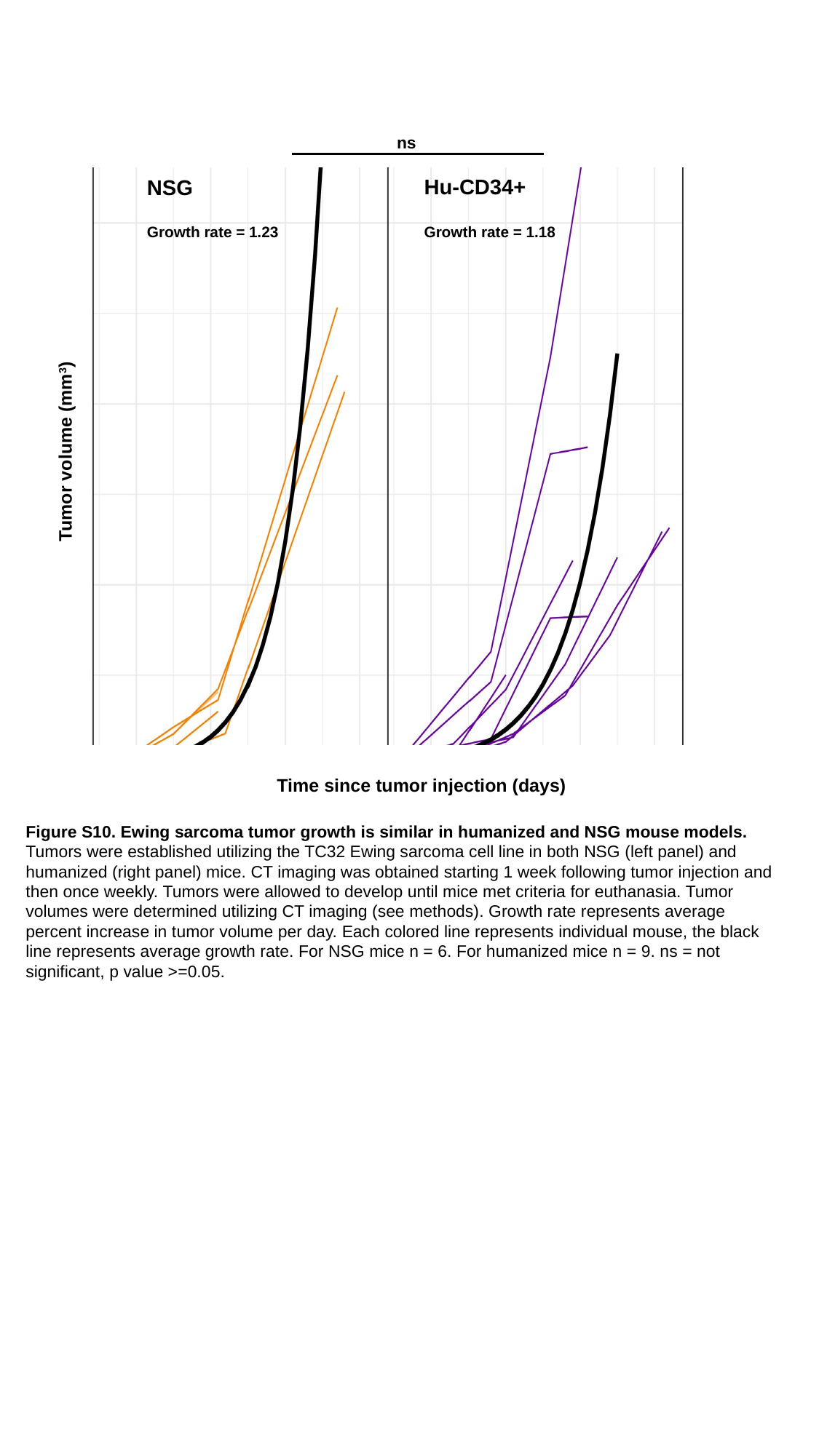

ns
Hu-CD34+
NSG
Growth rate = 1.23
Growth rate = 1.18
Tumor volume (mm3)
Time since tumor injection (days)
Figure S10. Ewing sarcoma tumor growth is similar in humanized and NSG mouse models. Tumors were established utilizing the TC32 Ewing sarcoma cell line in both NSG (left panel) and humanized (right panel) mice. CT imaging was obtained starting 1 week following tumor injection and then once weekly. Tumors were allowed to develop until mice met criteria for euthanasia. Tumor volumes were determined utilizing CT imaging (see methods). Growth rate represents average percent increase in tumor volume per day. Each colored line represents individual mouse, the black line represents average growth rate. For NSG mice n = 6. For humanized mice n = 9. ns = not significant, p value >=0.05.
