# Supplementary material for: TGFβ Inhibition during Radiotherapy Enhances Immune Cell Infiltration and Decreases Metastases in Ewing Sarcoma
Source: Cancer Res Commun. 2025 Aug 27;5(8):1441–57. doi: 10.1158/2767-9764.CRC-24-0346 (PMC12380665; doi:10.1158/2767-9764.CRC-24-0346)
Supplement: Figure S11 — Distinct tumor transcriptional signatures following radiation therapy are noted in Ewing sarcomas developed in hu-cD34+ versus NSG mouse models. [file crc-24-0346_figure_s11_suppsf11.pptx]

## Slide 1
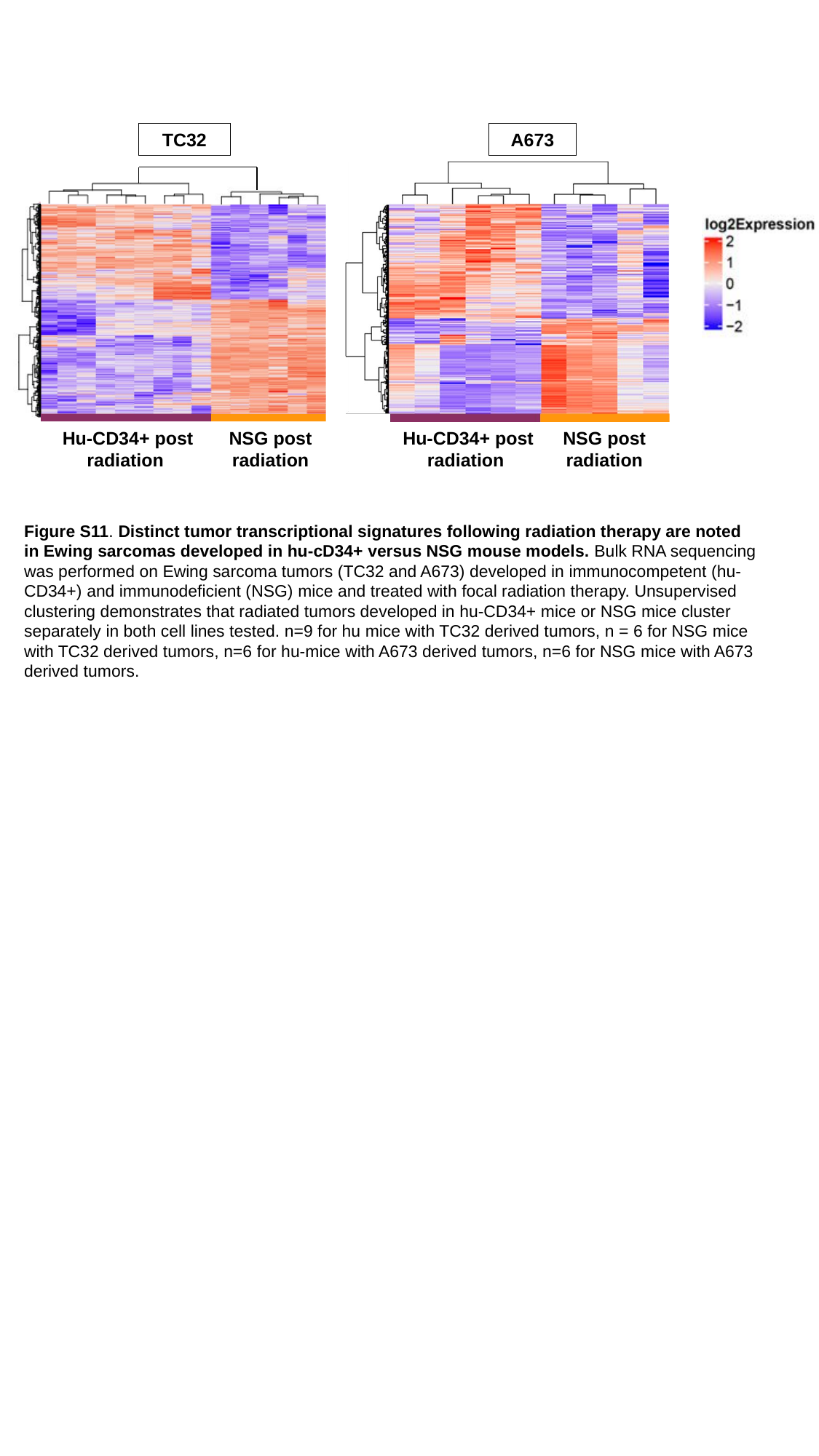

TC32
A673
Hu-CD34+ post
radiation
NSG post
radiation
Hu-CD34+ post
radiation
NSG post
radiation
Figure S11. Distinct tumor transcriptional signatures following radiation therapy are noted in Ewing sarcomas developed in hu-cD34+ versus NSG mouse models. Bulk RNA sequencing was performed on Ewing sarcoma tumors (TC32 and A673) developed in immunocompetent (hu-CD34+) and immunodeficient (NSG) mice and treated with focal radiation therapy. Unsupervised clustering demonstrates that radiated tumors developed in hu-CD34+ mice or NSG mice cluster separately in both cell lines tested. n=9 for hu mice with TC32 derived tumors, n = 6 for NSG mice with TC32 derived tumors, n=6 for hu-mice with A673 derived tumors, n=6 for NSG mice with A673 derived tumors.
