# Supplementary material for: TGFβ Inhibition during Radiotherapy Enhances Immune Cell Infiltration and Decreases Metastases in Ewing Sarcoma
Source: Cancer Res Commun. 2025 Aug 27;5(8):1441–57. doi: 10.1158/2767-9764.CRC-24-0346 (PMC12380665; doi:10.1158/2767-9764.CRC-24-0346)
Supplement: Figure S12 — Transcriptional modulation induced following radiation in A673 Ewing sarcoma tumors developed in hu-CD34+ versus NSG mice. [file crc-24-0346_figure_s12_suppsf12.pptx]

## Slide 1
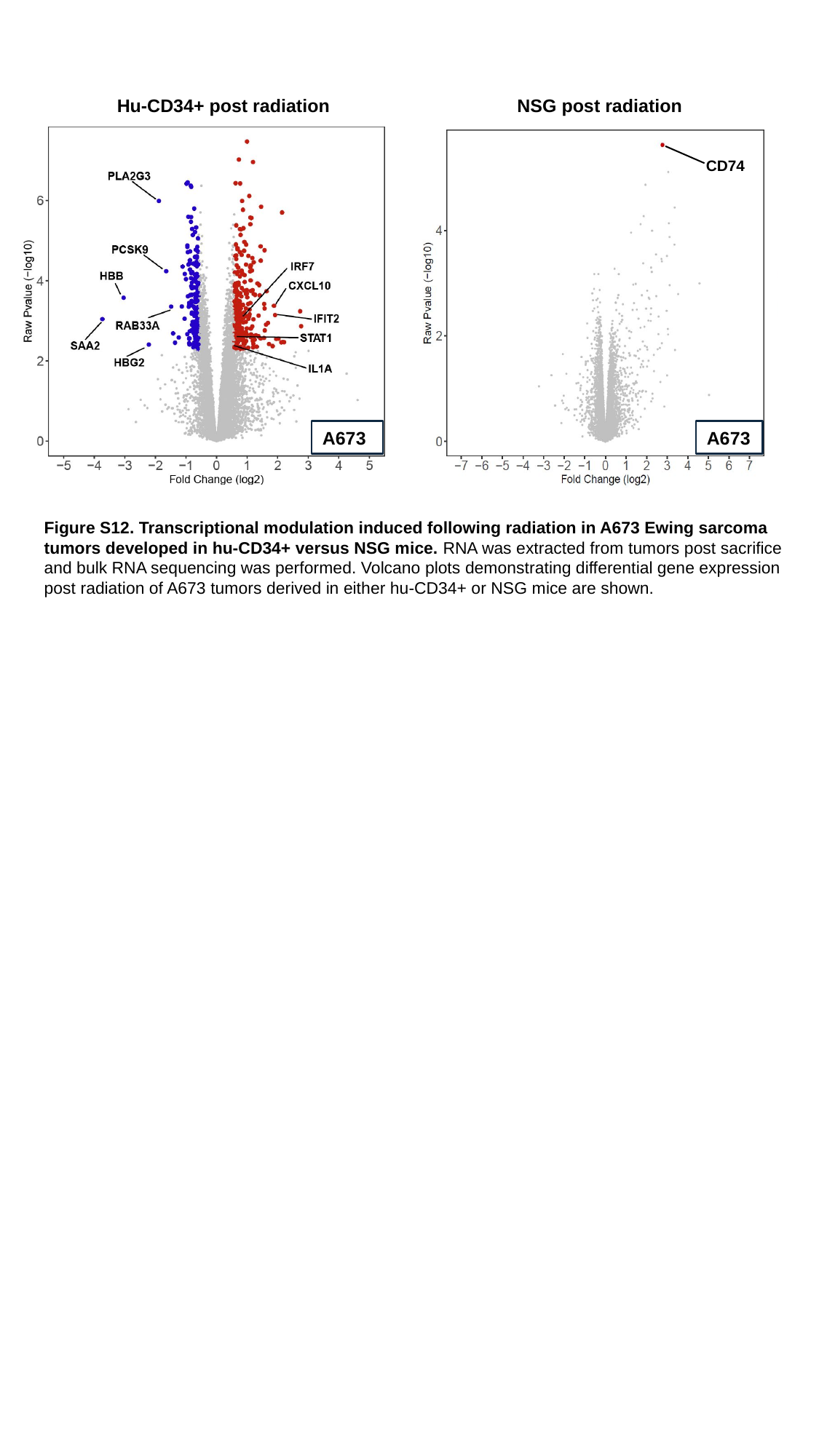

Hu-CD34+ post radiation
NSG post radiation
CD74
A673
A673
Figure S12. Transcriptional modulation induced following radiation in A673 Ewing sarcoma tumors developed in hu-CD34+ versus NSG mice. RNA was extracted from tumors post sacrifice and bulk RNA sequencing was performed. Volcano plots demonstrating differential gene expression post radiation of A673 tumors derived in either hu-CD34+ or NSG mice are shown.
