# Supplementary material for: TGFβ Inhibition during Radiotherapy Enhances Immune Cell Infiltration and Decreases Metastases in Ewing Sarcoma
Source: Cancer Res Commun. 2025 Aug 27;5(8):1441–57. doi: 10.1158/2767-9764.CRC-24-0346 (PMC12380665; doi:10.1158/2767-9764.CRC-24-0346)
Supplement: Figure S13 — Radiation therapy induces upregulation of inflammatory pathways in Ewing sarcoma tumors developed in a hu-CD34+ mouse model. [file crc-24-0346_figure_s13_suppsf13.pptx]

## Slide 1
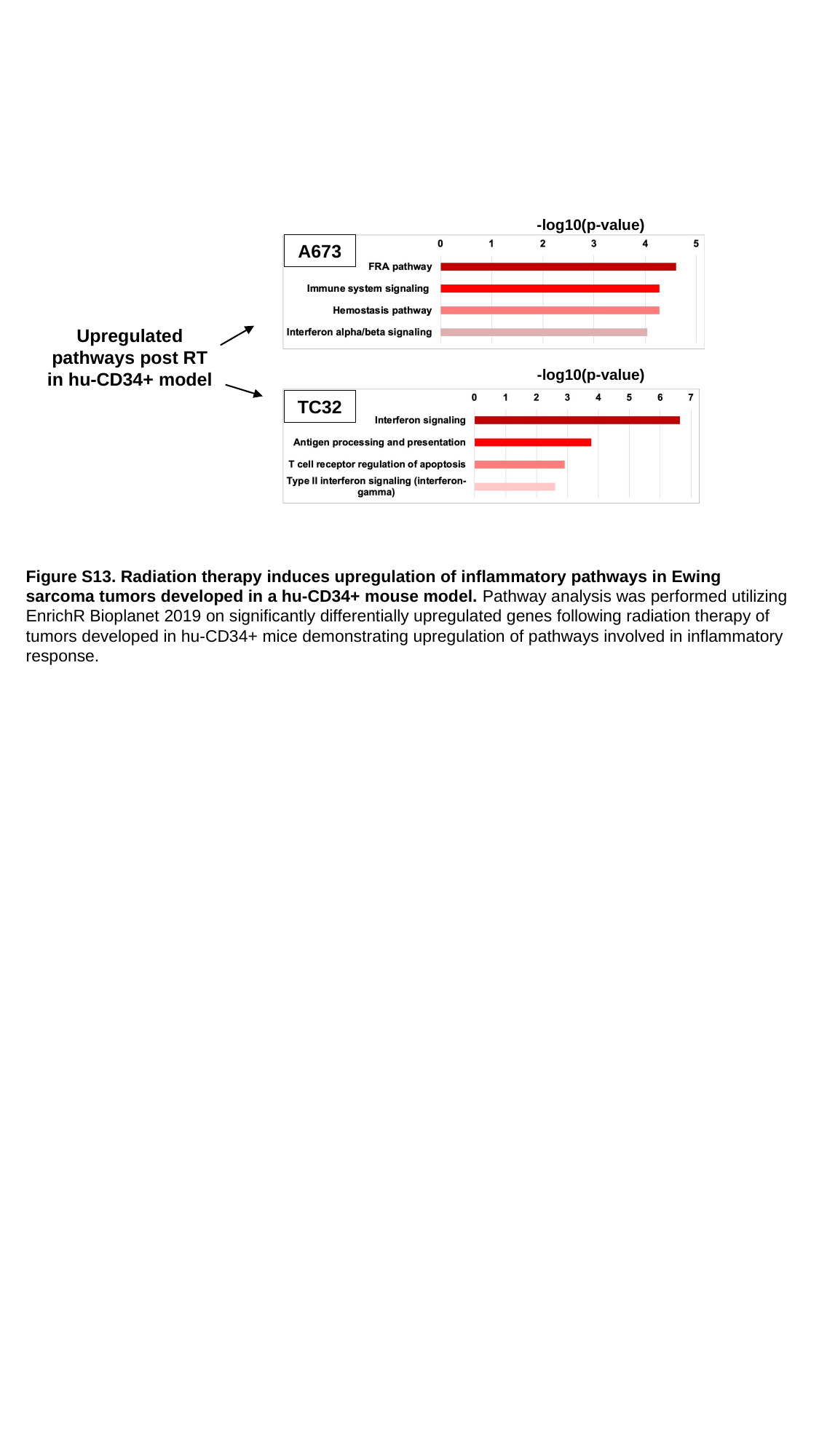

-log10(p-value)
A673
Upregulated pathways post RT in hu-CD34+ model
-log10(p-value)
TC32
Figure S13. Radiation therapy induces upregulation of inflammatory pathways in Ewing sarcoma tumors developed in a hu-CD34+ mouse model. Pathway analysis was performed utilizing EnrichR Bioplanet 2019 on significantly differentially upregulated genes following radiation therapy of tumors developed in hu-CD34+ mice demonstrating upregulation of pathways involved in inflammatory response.
