# Supplementary material for: TGFβ Inhibition during Radiotherapy Enhances Immune Cell Infiltration and Decreases Metastases in Ewing Sarcoma
Source: Cancer Res Commun. 2025 Aug 27;5(8):1441–57. doi: 10.1158/2767-9764.CRC-24-0346 (PMC12380665; doi:10.1158/2767-9764.CRC-24-0346)
Supplement: Figure S14 — The TC32 EwS cell line demonstrates increased sensitivity to radiation therapy compared to the A673 cell line in vitro. [file crc-24-0346_figure_s14_suppsf14.pptx]

## Slide 1
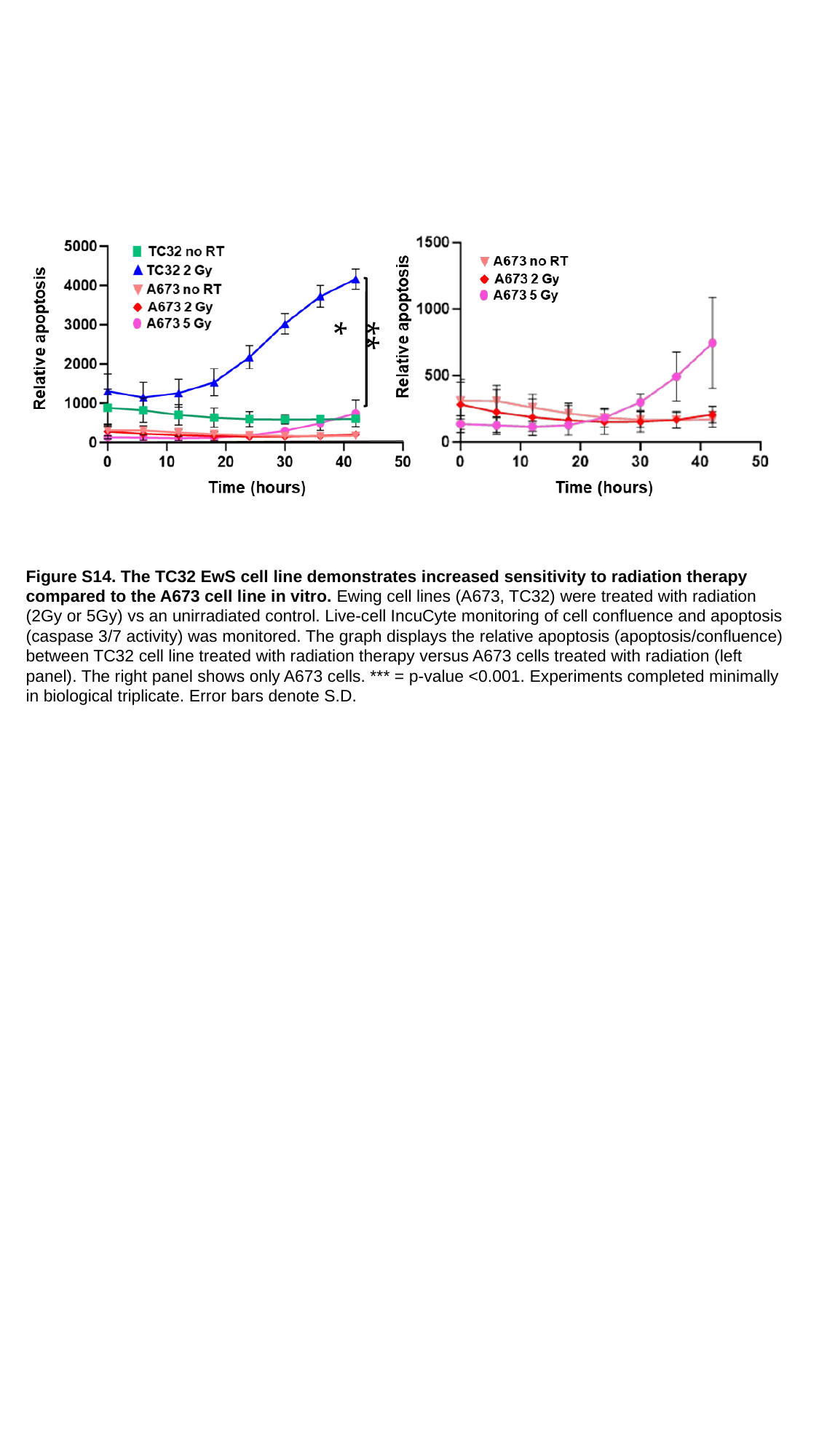

***
Figure S14. The TC32 EwS cell line demonstrates increased sensitivity to radiation therapy compared to the A673 cell line in vitro. Ewing cell lines (A673, TC32) were treated with radiation (2Gy or 5Gy) vs an unirradiated control. Live-cell IncuCyte monitoring of cell confluence and apoptosis (caspase 3/7 activity) was monitored. The graph displays the relative apoptosis (apoptosis/confluence) between TC32 cell line treated with radiation therapy versus A673 cells treated with radiation (left panel). The right panel shows only A673 cells. *** = p-value <0.001. Experiments completed minimally in biological triplicate. Error bars denote S.D.
