# Supplementary material for: TGFβ Inhibition during Radiotherapy Enhances Immune Cell Infiltration and Decreases Metastases in Ewing Sarcoma
Source: Cancer Res Commun. 2025 Aug 27;5(8):1441–57. doi: 10.1158/2767-9764.CRC-24-0346 (PMC12380665; doi:10.1158/2767-9764.CRC-24-0346)
Supplement: Figure S15 — Flow cytometry gating strategy. [file crc-24-0346_figure_s15_suppsf15.pptx]

## Slide 1
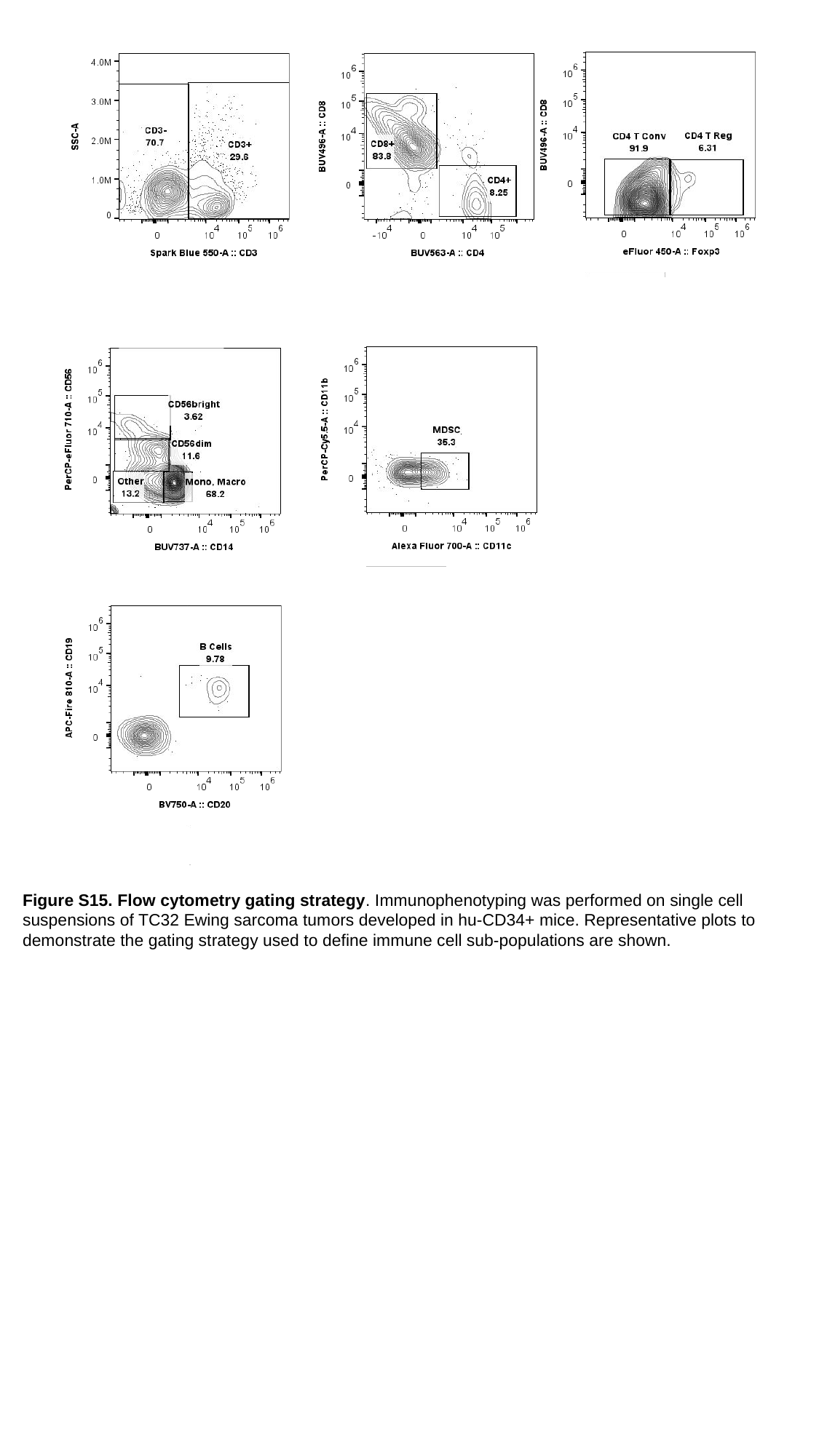

Figure S15. Flow cytometry gating strategy. Immunophenotyping was performed on single cell suspensions of TC32 Ewing sarcoma tumors developed in hu-CD34+ mice. Representative plots to demonstrate the gating strategy used to define immune cell sub-populations are shown.
