# Supplementary material for: TGFβ Inhibition during Radiotherapy Enhances Immune Cell Infiltration and Decreases Metastases in Ewing Sarcoma
Source: Cancer Res Commun. 2025 Aug 27;5(8):1441–57. doi: 10.1158/2767-9764.CRC-24-0346 (PMC12380665; doi:10.1158/2767-9764.CRC-24-0346)
Supplement: Figure S16 — Survival of mice treated with ± RER and ± radiation therapy. [file crc-24-0346_figure_s16_suppsf16.pptx]

## Slide 1
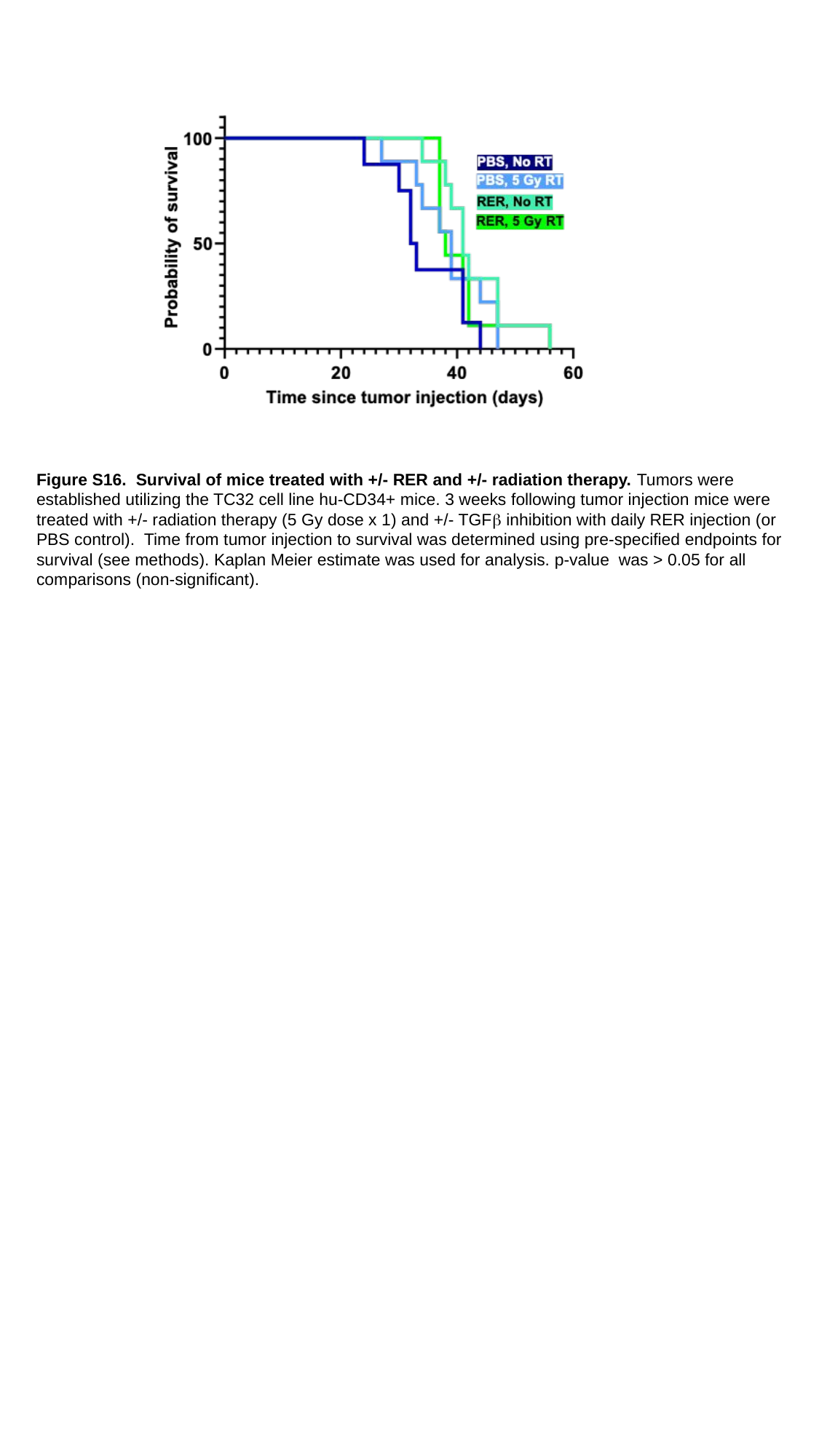

Figure S16. Survival of mice treated with +/- RER and +/- radiation therapy. Tumors were established utilizing the TC32 cell line hu-CD34+ mice. 3 weeks following tumor injection mice were treated with +/- radiation therapy (5 Gy dose x 1) and +/- TGFβ inhibition with daily RER injection (or PBS control). Time from tumor injection to survival was determined using pre-specified endpoints for survival (see methods). Kaplan Meier estimate was used for analysis. p-value was > 0.05 for all comparisons (non-significant).
