# Supplementary material for: TGFβ Inhibition during Radiotherapy Enhances Immune Cell Infiltration and Decreases Metastases in Ewing Sarcoma
Source: Cancer Res Commun. 2025 Aug 27;5(8):1441–57. doi: 10.1158/2767-9764.CRC-24-0346 (PMC12380665; doi:10.1158/2767-9764.CRC-24-0346)
Supplement: Figure S17 — EWS::FLI1 expression in tumors established in hu-mice. [file crc-24-0346_figure_s17_suppsf17.pptx]

## Slide 1
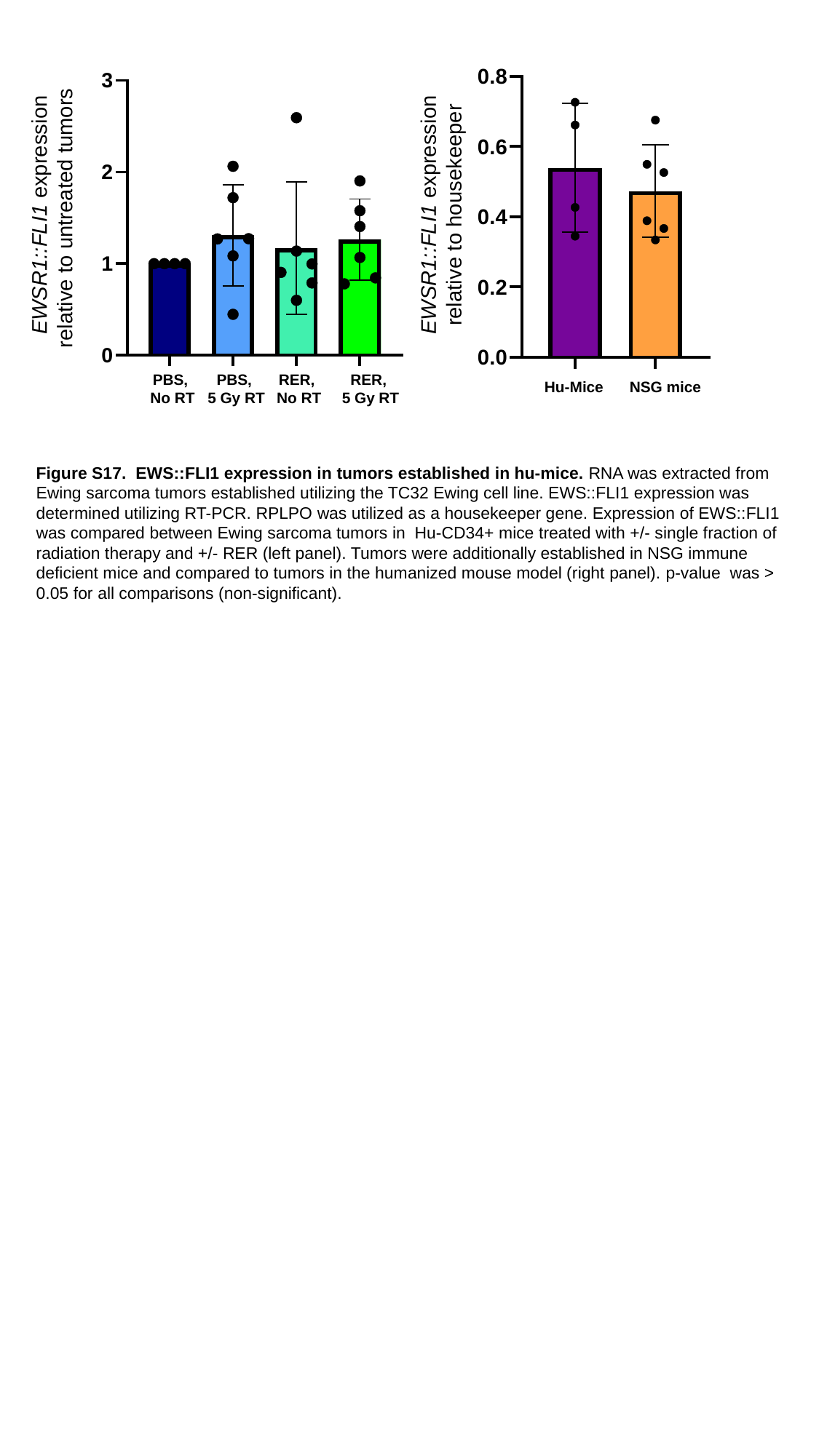

EWSR1::FLI1 expression relative to housekeeper
EWSR1::FLI1 expression relative to untreated tumors
PBS,
 No RT
PBS,
5 Gy RT
RER,
 No RT
RER,
 5 Gy RT
Hu-Mice
NSG mice
Figure S17. EWS::FLI1 expression in tumors established in hu-mice. RNA was extracted from Ewing sarcoma tumors established utilizing the TC32 Ewing cell line. EWS::FLI1 expression was determined utilizing RT-PCR. RPLPO was utilized as a housekeeper gene. Expression of EWS::FLI1 was compared between Ewing sarcoma tumors in Hu-CD34+ mice treated with +/- single fraction of radiation therapy and +/- RER (left panel). Tumors were additionally established in NSG immune deficient mice and compared to tumors in the humanized mouse model (right panel). p-value was > 0.05 for all comparisons (non-significant).
