# Supplementary material for: TGFβ Inhibition during Radiotherapy Enhances Immune Cell Infiltration and Decreases Metastases in Ewing Sarcoma
Source: Cancer Res Commun. 2025 Aug 27;5(8):1441–57. doi: 10.1158/2767-9764.CRC-24-0346 (PMC12380665; doi:10.1158/2767-9764.CRC-24-0346)
Supplement: Figure S18 — Representative H&E images of mouse lungs used for metastatic quantification. [file crc-24-0346_figure_s18_suppsf18.pptx]

## Slide 1
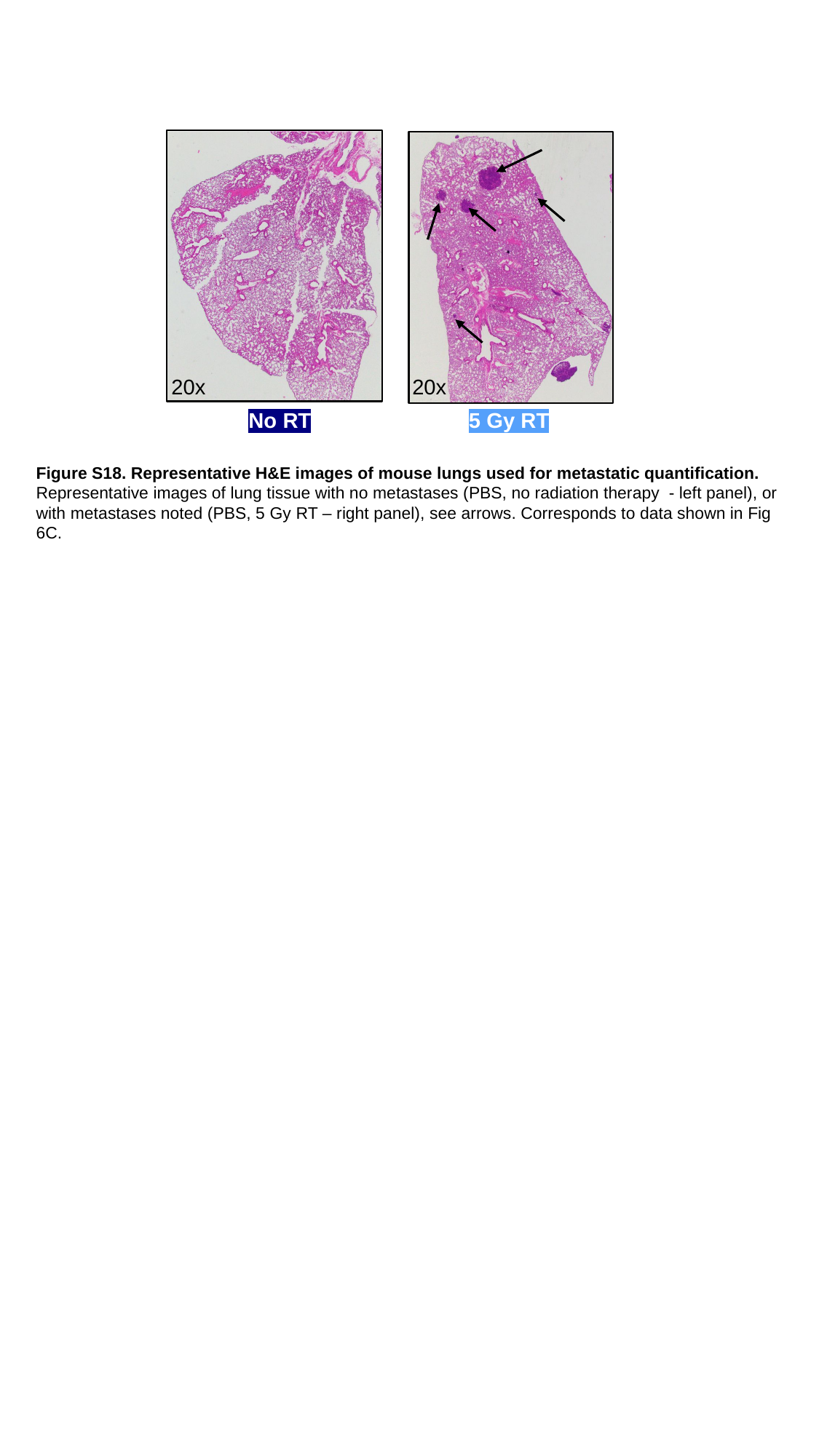

20x
20x
No RT
5 Gy RT
Figure S18. Representative H&E images of mouse lungs used for metastatic quantification. Representative images of lung tissue with no metastases (PBS, no radiation therapy - left panel), or with metastases noted (PBS, 5 Gy RT – right panel), see arrows. Corresponds to data shown in Fig 6C.
