# Supplementary material for: TGFβ Inhibition during Radiotherapy Enhances Immune Cell Infiltration and Decreases Metastases in Ewing Sarcoma
Source: Cancer Res Commun. 2025 Aug 27;5(8):1441–57. doi: 10.1158/2767-9764.CRC-24-0346 (PMC12380665; doi:10.1158/2767-9764.CRC-24-0346)
Supplement: Figure S19 — TGFβ signaling is decreased in tumors of mice that receive RER following radiation therapy as compared to mice that are treated with radiation therapy (RT) alone. [file crc-24-0346_figure_s19_suppsf19.pptx]

## Slide 1
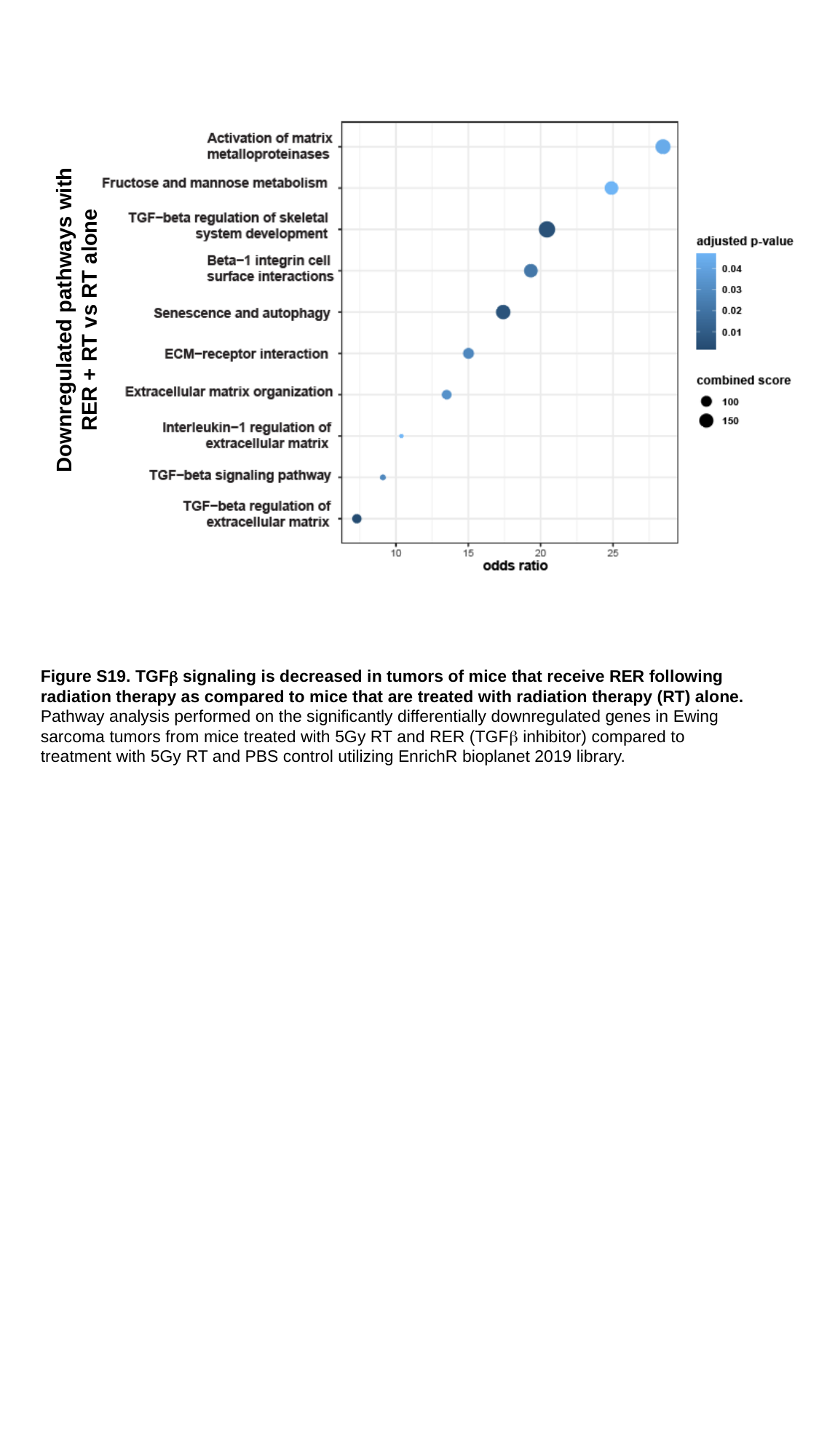

Downregulated pathways with RER + RT vs RT alone
Figure S19. TGFβ signaling is decreased in tumors of mice that receive RER following radiation therapy as compared to mice that are treated with radiation therapy (RT) alone. Pathway analysis performed on the significantly differentially downregulated genes in Ewing sarcoma tumors from mice treated with 5Gy RT and RER (TGFβ inhibitor) compared to treatment with 5Gy RT and PBS control utilizing EnrichR bioplanet 2019 library.
