# Supplementary material for: TGFβ Inhibition during Radiotherapy Enhances Immune Cell Infiltration and Decreases Metastases in Ewing Sarcoma
Source: Cancer Res Commun. 2025 Aug 27;5(8):1441–57. doi: 10.1158/2767-9764.CRC-24-0346 (PMC12380665; doi:10.1158/2767-9764.CRC-24-0346)
Supplement: Table S2 — Sample characteristics [file crc-24-0346_table_s2_suppst2.docx]

| Sex | Site of biopsy | Point in therapy | Disease site of initial diagnosis | Cells sequenced | GEO reference number |
| --- | --- | --- | --- | --- | --- |
| M | Lung | 1^st^ relapse | Lower extremity | CD45+  Cells only | GSE198896 |
| F | Soft tissue | Diagnosis | Abdominal wall | CD45+ cells only | GSE198896 |
| M | Lung/chest | 2^nd^ relapse | Paraspinal | CD45+ cells only | GSE198896 |
| M | Lower extremity | Diagnosis | Lower extremity | CD45+ cells only | GSE198896 |
| UA | Extremity | Diagnosis | Localized extremity | All cells | GSE261693 |
| M | Clavicle | Local control | Metastatic: clavicle and lungs | All cells | GSE261693 |
| F | Upper extremity | Diagnosis | Humerus | All cells | GSE261693 |

**Table S2**. **Ewing tumor single cell RNA sequencing samples patient characteristics and sample information**. A total of 4 human Ewing tumors underwent cell sorting utilizing flow cytometry to isolate CD45+ cells. Single cell RNA sequencing was performed on the CD45+ cell isolated from these 4 human Ewing samples. 3 additional human Ewing tumors underwent single cell RNA sequencing of all cell types present in the sample. This table is modified from Supplementary Table 1 in Cillo et al (22).
