# Supplementary material for: TGFβ Inhibition during Radiotherapy Enhances Immune Cell Infiltration and Decreases Metastases in Ewing Sarcoma
Source: Cancer Res Commun. 2025 Aug 27;5(8):1441–57. doi: 10.1158/2767-9764.CRC-24-0346 (PMC12380665; doi:10.1158/2767-9764.CRC-24-0346)
Supplement: Table S4 — immune cell infiltrates in human tumors [file crc-24-0346_table_s4_suppst4.docx]

| **Tumor type** | **%viable CD45+ (PE) cells** |
| --- | --- |
| Ewing sarcoma | 11.8 |
| osteosarcoma | 33.7 |

**Table S3. Examples of % viable CD45+ cells from patient tumor biopsy specimens.** 100-200mg tumor tissue from biopsy specimens (IRB approved STUDY20010034, as previously described by Cillo et al., PMID 36074145) were analyzed by flow cytometry for viable, CD45+ cells. One Ewing sarcoma and one osteosarcoma example are included. Of note, biopsy specimens may not be representative of all regions of the tumor.
